# Molecular control of PDPN^hi^ macrophage subset induction by ADAP as a host defense in sepsis

**DOI:** 10.1172/jci.insight.186456

**Published:** 2025-02-04

**Authors:** Pengchao Zhang, Xinning Wang, Xiaodong Yang, Hebin Liu

**Affiliations:** 1 MOE Key Laboratory of Geriatric Diseases and Immunology, Institutes of Biology and Medical Sciences, Suzhou Medical College of Soochow University, Soochow University, Suzhou, Jiangsu Province, China.; 2 Department of Gastrointestinal Surgery, The Second Affiliated Hospital of Soochow University, Suzhou, Jiangsu Province, China.

**Keywords:** Immunology, Inflammation, Bacterial infections, Macrophages, Signal transduction

## Abstract

Induction of podoplanin (PDPN) expression is a critical response of macrophages to LPS stimulation or bacterial infection in sepsis, but how this key process of TLR4-stimulated PDPN upregulation is regulated and the effect of PDPN expression on macrophage function remain elusive. Here, we determined how this process is regulated in vitro and in vivo. PDPN failed to be upregulated in TLR4-stimulated macrophages deficient in adhesion and degranulation-promoting adapter protein (ADAP), which could be rescued by the reconstitution of ADAP. A distinct PDPN^hi^ peritoneal macrophage (PM) subset, which exhibited an M2-like phenotype and enhanced phagocytic activity, was generated in WT but not in ADAP-deficient septic mice. The blockade of PDPN^hi^ PMs mimicked the effect of ADAP deficiency, which exacerbated sepsis. Mechanistically, Bruton’s tyrosine kinase–mediated (BTK-mediated) tyrosine phosphorylation of ADAP at Y^571^ worked together with mTOR to converge on STAT3 activation for the transactivation of the PDPN promoter. Moreover, agonist activation of STAT3 profoundly potentiated the PDPN^hi^ PM subset generation and alleviated sepsis severity in mice. Together, our findings reveal a mechanism whereby ADAP resets macrophage function by controlling the TLR4-induced upregulation of PDPN as a host innate immune defense during sepsis.

## Abstract 2

ADAP regulates sepsis progression by controlling the TLR4-induced upregulation of PDPN to form a PDPN^hi ^macrophage subpopulation, which exerts enhanced antibacterial functions during sepsis.

## Graphical abstract

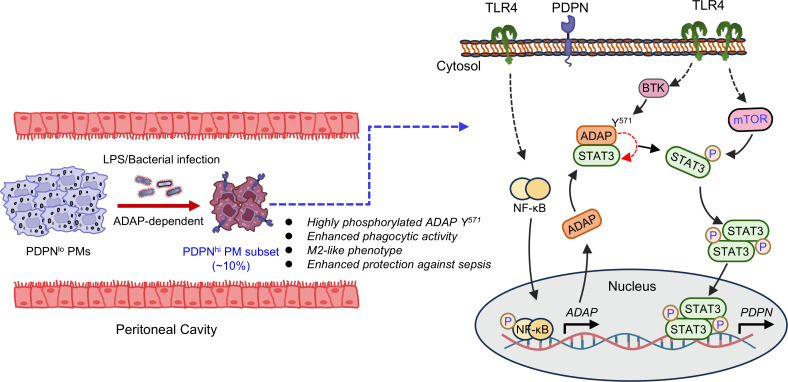

## Introduction

Sepsis is a life-threatening organ dysfunction caused by dysregulated host response to infection (1). It is estimated that there were 48.9 million patients with sepsis and 11 million sepsis-related deaths worldwide in 2017, accounting for 19.7% of the total global deaths (2). Sepsis is primarily caused by bacterial infections but can also be caused by fungi, parasites, and viruses; for example, sepsis is also a common complication observed in severe cases of COVID-19 (3, 4). Sepsis remains one of the most pressing public health burdens worldwide (1, 5).

Adhesion and degranulation-promoting adapter protein (ADAP), also known as Fyn-binding protein (FYB), is a hematopoietic cell–specific immune adapter protein (6). While originally defined as a T cell adaptor central for T cell receptor and integrin signaling for T cell adhesion (7, 8), ADAP also functions in a variety of innate immune cells, including macrophages, mast cells, and DCs (9–11). The macrophage, a versatile sentinel immune cell, plays a central role in immune homeostasis and host defense against invading microbes (12, 13). Our previous study showed that ADAP deficiency potentiates macrophage M1 polarization and the inflammatory response upon LPS stimulation (14). ADAP restrains Fcγ receptor I/IV transcription and mediates the phagocytic ability of splenic macrophages by interacting with STAT1 in immune thrombocytopenia (15). Despite these findings, the functional role of ADAP in macrophage-mediated host innate immune defense during sepsis remains unclear.

Podoplanin (PDPN), a highly conserved mucin-type transmembrane protein, is the only known endogenous ligand for C-type lectin-like receptor 2 (CLEC-2). The central physiological role of PDPN is to aggregate and activate platelets that highly express CLEC-2 (16, 17). PDPN is expressed in the nonhematopoietic cell types such as fibroblasts and epithelial cells (18–21), and it is rarely found in resting macrophages but is highly expressed in LPS-treated inflammatory macrophages, usually associated with inflammation or cancer (17, 22–27). In sepsis, the interaction between macrophage PDPN and platelet CLEC-2 is critical for recruiting macrophages to the infection site to promote bacterial clearance (23). The platelet CLEC-2 prevents excessive infiltration and accumulation of macrophages in the peritoneal cavity, limiting the series of organ damages caused by excessive inflammation during murine peritonitis (24). Furthermore, the PDPN^+^ macrophages in the spleen exhibit marked phagocytic potential and elevated numbers in mice following systemic zymosan treatment (25). This evidence suggests that PDPN acts as a TLR4 ligand–induced cell surface protein, and its upregulation in macrophages is a key event in the macrophage response to bacterial infections during sepsis. Despite these findings, it remains obscure how LPS-induced PDPN expression in macrophages is precisely regulated and what the functional role of the induced PDPN^+^ macrophage subpopulation is during inflammatory diseases such as sepsis.

In this study, we report the discovery of a mechanism regulating LPS-induced PDPN expression to form a PDPN^+^ macrophage population by ADAP, an immune adaptor protein. In a sepsis mouse model, ADAP is indispensable for the induction of the PDPN^hi^ peritoneal macrophage (PM) subset, which exhibits enhanced phagocytic activity; is closely akin to an M2 phenotype; and is required for the control of sepsis severity. Mechanistically, Bruton’s tyrosine kinase–mediated (BTK-mediated) tyrosine phosphorylation of ADAP at Y^571^, together with mTOR-mediated phosphorylation, converges with STAT3 activation for the transactivation of PDPN transcription in macrophages.. Thus, ADAP provides molecular control in the pathogenesis of sepsis by regulating PDPN expression in PMs, and correction of ADAP phosphorylation and induction of the PDPN^hi^ PM subset could provide treatment strategies for sepsis.

## Results

### ADAP is an LPS stimulus–responsive protein in macrophages, and its expression is associated with sepsis.

**Figure 1:**
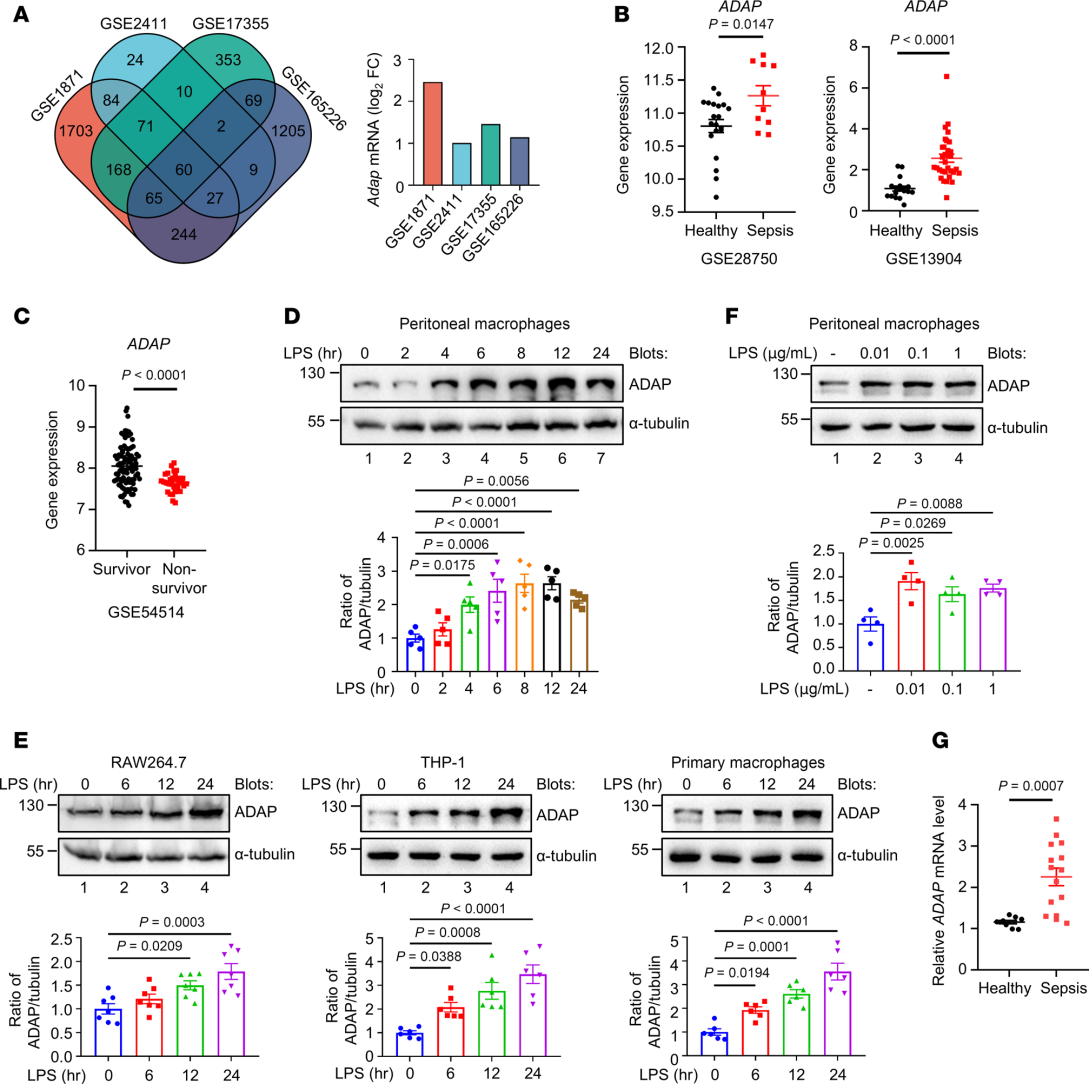
ADAP is an LPS stimulus–responsive protein in macrophages, and its expression is associated with sepsis. (**A**) Left panel: Venn diagram of the DEGs in all 4 GEO datasets (GSE1871, GSE2411, GSE17355, and GSE165226) from the lung tissues of sepsis mouse models. Shared DEGs were identified (log_2_[fold change] ≤ −1/ ≥ 1, adj. *P* < 0.05). Right panel: Relative *Adap* expression in the lungs of these datasets, analyzed using GEO2R. (**B**) *ADAP* mRNA expression in patients with sepsis versus healthy controls in GSE datasets GSE28750 (healthy, *n* = 20; sepsis, *n* = 10) and GSE13904 (healthy, *n* = 18; sepsis, *n* = 32), analyzed using GEO2R (unpaired *t* test). (**C**) *ADAP* mRNA levels in septic survivors versus nonsurvivors from GSE54514 (survivor, *n* = 95; nonsurvivor, *n* = 31), analyzed using GEO2R (unpaired *t* test). (**D** and **E**) ADAP protein expression in mouse PMs (**D**), RAW264.7 cells (**E**, left panel), THP-1 cells (**E**, middle panel), and primary human macrophages (**E**, right panel) mock-treated or stimulated with LPS (100 ng/mL) for indicated times, assessed by Western blot. Bar charts show densitometry analysis of ADAP normalized to α-tubulin (PMs, *n* = 5; RAW264.7 cells, *n* = 7; THP-1 cells, *n* = 6; primary human macrophages, *n* = 6; 1-way ANOVA, Tukey’s multiple-comparison test). (**F**) ADAP expression in PMs treated with increasing doses of LPS (0.01, 0.1, and 1 μg/mL) for 24 hours was analyzed by Western blotting. Bar chart shows densitometry analysis of ADAP normalized to α-tubulin (*n* = 4 each, 1-way ANOVA, Tukey’s multiple-comparison test). (**G**) *ADAP* mRNA expression in the peripheral blood mononuclear cells (PBMC) of patients with sepsis versus healthy controls was measured by qPCR (healthy, *n* = 9; sepsis, *n* = 15; unpaired *t* test). Relative mRNA levels were normalized to *Ywhaz*.

To mine key genes critical for sepsis, we cross-analyzed 4 gene expression profile databases (GSE1871, GSE2411, GSE17355, and GSE165226) from the lung tissues of sepsis mouse models. As shown in Figure 1A, Venn diagram analysis identified 60 shared differentially expressed genes (DEGs) among the 4 sepsis datasets, among which *Fyb*, a gene encoding ADAP of hematopoietic adapter protein, was upregulated in all 4 databases (Figure 1A, right panel), suggesting a link between ADAP expression and sepsis. Furthermore, analysis of the biological information data of patients with sepsis in the Gene Expression Omnibus (GEO) database showed that *ADAP* expression was significantly higher in the peripheral blood of patients with sepsis than in healthy controls (Figure 1B). Despite this, of note, *ADAP* was significantly downregulated in patients who died of sepsis (Figure 1C), suggesting that in instances of severe sepsis, the underexpression of ADAP is associated with sepsis severity and poor prognosis. Furthermore, we investigated whether ADAP expression is regulated by LPS stimulation in macrophages. As shown in Figure 1, D and E, the expression of ADAP was significantly increased in response to LPS stimulation in a time-dependent manner in various macrophage cells such as primary mouse PMs, mouse macrophage cell line RAW264.7 cells, human THP-1–derived macrophages, and primary human macrophages. Stimulation with different doses of LPS showed that low doses of LPS could significantly enhance the expression of ADAP in mouse PMs (Figure 1F). Thus, ADAP is an LPS stimulus–responsive protein in macrophages. Moreover, analysis complementary to those above with clinical blood samples from patients with sepsis or healthy individuals showed that the relative levels of *ADAP* expression were significantly increased in patients with sepsis (Figure 1G). These findings demonstrate that ADAP expression is induced in LPS-stimulated macrophages and is associated with sepsis severity.

### ADAP deficiency is associated with increased inflammation and disease severity in sepsis.

**Figure 2:**
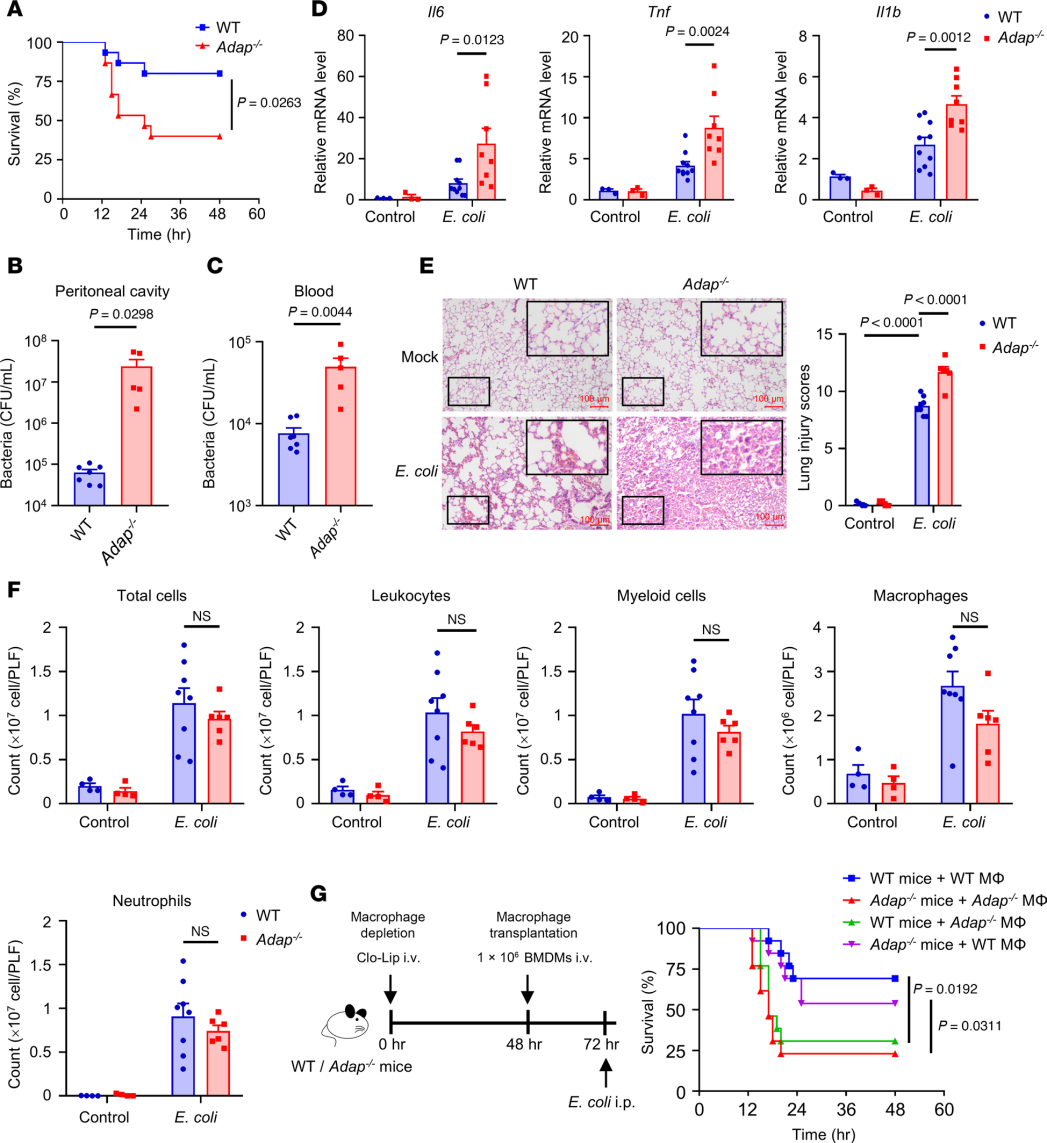
ADAP deficiency is associated with increased inflammation and disease severity in sepsis. (**A**) Kaplan-Meier survival curves of WT and *Adap^–/–^* mice challenged with *E*. *coli* (2 × 10^7^ CFU, i.p.) (*n* = 15 each, log-rank test). (**B** and **C**) Bacterial burden in the peritoneal lavage fluid (**B**) and blood (**C**) of WT and *Adap^–/–^* septic mice 18 hours after injection of saline or *E*. *coli* (WT *E*. *coli*, *n* = 7; *Adap^–/–^* *E*. *coli*, *n* = 5; unpaired *t* test). (**D**) mRNA levels of *Il6*, *Tnf*, and *Il1b* in lung tissues of WT and *Adap^–/–^* mice 18 hours after i.p. injection of saline or *E*. *coli* (WT saline or *Adap^–/–^* saline, *n* = 3; WT *E*. *coli*, *n* = 10; *Adap^–/–^* *E*. *coli*, *n* = 8; 2-way ANOVA, Šidák’s multiple-comparison test). Relative mRNA levels were normalized to *Gapdh*. (**E**) Representative H&E-stained lung sections of WT and *Adap^–/–^* mice 18 hours after *E*. *coli* injection. Scale bars: 100 μm. Lung injury was scored and compared between the groups (WT saline or *Adap^–/–^* saline, *n* = 4; WT *E*. *coli*, *n* = 8; *Adap^–/–^* *E*. *coli*, *n* = 6; 2-way ANOVA, Tukey’s multiple-comparison test). (**F**) Flow cytometric analysis of myeloid cells (CD45^+^CD11b^+^), macrophages (CD45^+^CD11b^+^F4/80^+^), and neutrophils (CD45^+^CD11b^+^Ly6G^+^) from leukocytes (CD45^+^) in the peritoneal lavage fluid of WT and *Adap^–/–^* septic mice (WT saline or *Adap^–/–^* saline, *n* = 4; WT *E*. *coli*, *n* = 8; *Adap^–/–^* *E*. *coli*, *n* = 6; 2-way ANOVA, Šidák’s multiple-comparison test). (**G**) Kaplan-Meier survival curves of *E*. *coli*–injected WT and *Adap^–/–^* mice transplanted with WT or *Adap^–/–^* BMDMs (*n* = 13 each, log-rank test).

To evaluate the role of upregulated ADAP in sepsis, sepsis mouse models of both WT and *Adap^–/–^* septic mice were generated by i.p. injection of *E*. *coli* (2 × 10^7^ CFU/mouse) for 18 hours as previously reported (28, 29). As shown in Figure 2A, *Adap^–/–^* septic mice exhibited accelerated mortality with significantly decreased survival rates relative to WT mice, indicating the critical role of ADAP in prolonging survival during bacterial sepsis. Furthermore, the bacterial loads in both peritoneal lavage fluid and blood from *Adap^–/–^* septic mice were significantly higher than those from WT control mice (Figure 2, B and C). We further examined and compared the inflammatory response and tissue damage in WT and *Adap^–/–^* septic mice. The levels of proinflammatory cytokines, including *Il6*, *Tnf*, and *Il1b*, were substantially elevated in the lung tissue of *Adap^–/–^* septic mice compared with those in the WT group (Figure 2D). When we examined the lungs, it was observed that, while septic induction by *E*. *coli* injection caused significant alveolar septal wall thickening, peribronchial inflammation, airway lumen leukocyte accumulation, and severe pneumonia in the lung tissues of both WT and *Adap^–/–^* septic mice, the degree of damage was more profound in the lung tissues of *Adap^–/–^* mice than in those of WT mice (Figure 2E). Thus, these data demonstrate that there is an association between ADAP expression and inflammation and disease severity in sepsis, and they demonstrate that ADAP deficiency or underexpression exacerbates sepsis by promoting an inflammatory response.

Given the importance of inflammatory responses at the primary infection site in sepsis, we sought to determine whether ADAP deficiency affects the recruitment of leukocytes to the peritoneum under both resting and septic conditions. As expected, ADAP deficiency had little effect on the counts of immune cells, including myeloid cells, macrophages, and neutrophils, which were robustly increased to a comparable level in the peritoneum following the injection of *E*. *coli* for sepsis induction in WT and *Adap^–/–^* mice (Figure 2F). This suggests that ADAP deficiency does not affect peritoneal leukocyte recruitment in septic mice.

Given that ADAP expression is upregulated by LPS stimulation in macrophages, we assessed whether macrophages are responsible for the exacerbated sepsis and inflammation observed in *Adap^–/–^* mice in response to *E*. *coli* challenge using an adoptive transfer assay. As shown in Figure 2G, adoptive transfer of bone marrow–derived macrophages (BMDMs) isolated from WT mice into *Adap^–/–^* septic mice ameliorated disease severity and progression compared with *Adap^–/–^* mice transplanted with *Adap^–/–^* BMDMs. In contrast, the survival rate of WT septic mice decreased significantly when transplanted with *Adap^–/–^* BMDMs compared with those receiving WT BMDMs. These data suggest that ADAP expression in macrophages is responsible for the ADAP deficiency–mediated aggravation of sepsis.

### ADAP is indispensable for the induction of PDPN expression in macrophages in response to LPS stimulation or bacterial infection during sepsis.

**Figure 3:**
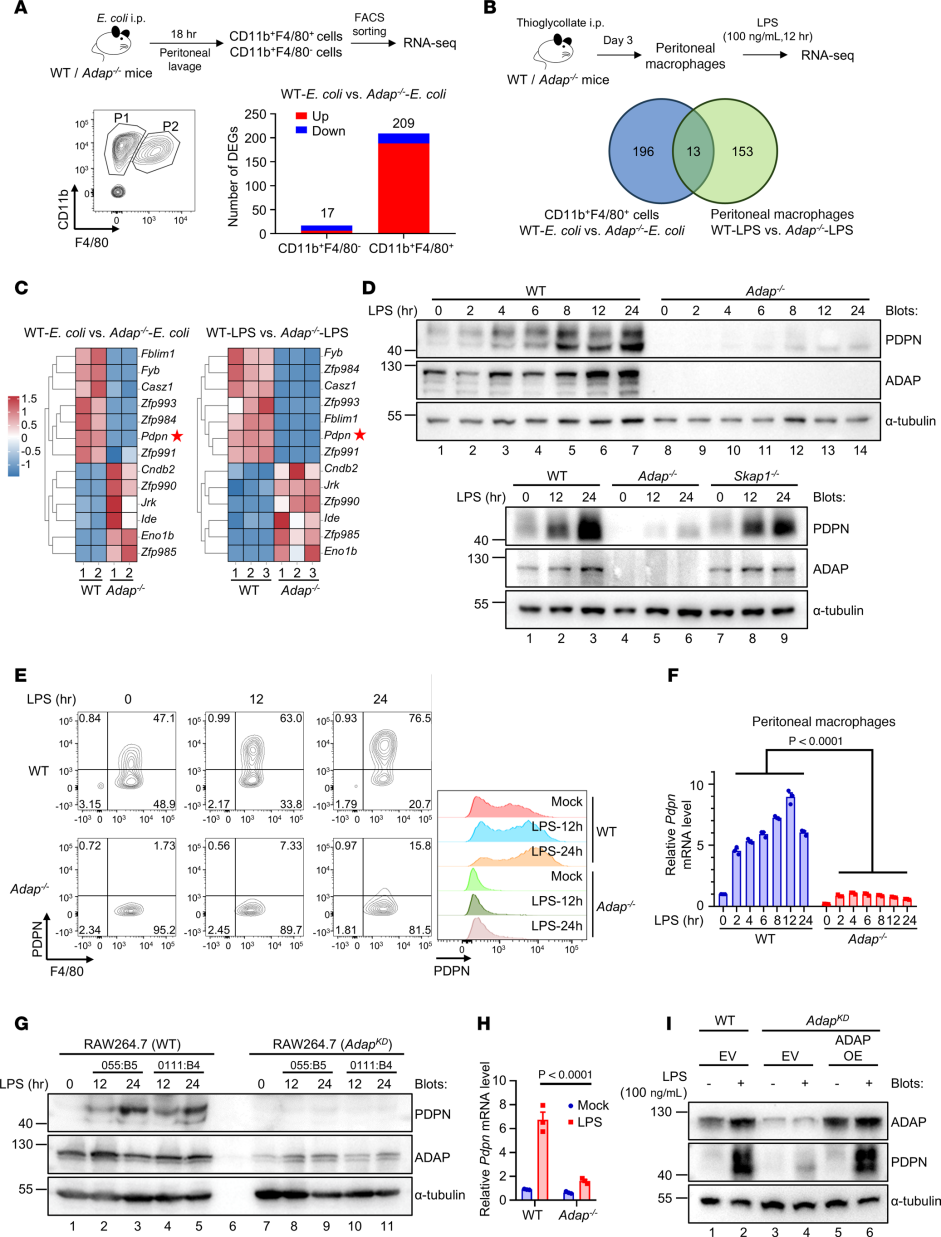
ADAP is indispensable for the induction of PDPN expression in macrophages in response to LPS stimulation or bacterial infection during sepsis. (**A**) Gating strategy for FACS of CD11b^+^F4/80^+^ and CD11b^+^F4/80^–^ cells from the peritoneal cells of WT and *Adap^–/–^* mice 18 hours after *E*. *coli* injection (2 × 10^7^ CFU, i.p.). RNA-Seq analysis identified DEGs (log_2_[fold change] ≤ −1/ ≥ 1, 5% FDR) in sorted cell populations. (**B**) Schematic of RNA-Seq analysis of WT and *Adap^–/–^* PMs exposed to LPS (100 ng/mL) for 12 hours in vitro (upper panel). Venn diagram of DEGs in WT versus *Adap^–/–^* PMs from *E. coli*–infected mice (209 DEGs) and WT versus *Adap^–/–^* PMs treated with LPS (166 DEGs), revealing 13 overlapping DEGs (lower panel). (**C**) Heatmap and hierarchical clustering of the 13 overlapping DEGs from **B**. (**D**–**F**) PDPN expression in WT and *Adap^–/–^* PMs with or without LPS stimulation (100 ng/mL) was analyzed by Western blotting (**D**), flow cytometry (**E**), and qPCR (**F**, *n* = 3 each, 2-way ANOVA, Šidák’s multiple-comparison test). Relative mRNA levels were normalized to *Hprt*. (**G**) Immunoblot analysis of PDPN in WT or ADAP-knockdown (ADAP^KD^) RAW264.7 cells stimulated with or without LPS (100 ng/mL) for the indicated time points. (**H**) *Pdpn* mRNA expression in WT and *Adap^–/–^* iBMMs with or without LPS stimulation (100 ng/mL, 12 hours), analyzed by qPCR (*n* = 3 each, 2-way ANOVA, Šidák’s multiple-comparison test). Relative mRNA levels were normalized to *Hprt*. (**I**) ADAP^KD^ RAW264.7 cells were reconstituted via lentiviral transduction with ADAP to induce overexpression (OE) or with an empty vector (EV) as a control, followed by mock treatment or stimulation with LPS (100 ng/mL) for 24 hours. Whole cell lysates were prepared and subjected to Western blot analysis of PDPN. α-Tubulin blots are derived from the same samples run contemporaneously in parallel gels.

Given the LPS-inducible upregulation of ADAP in macrophages and the association of ADAP expression level with aggravated sepsis, we explored the exact downstream target genes regulated by ADAP in macrophages in response to LPS stimulation and bacterial challenges. To this end, peritoneal cavity cells isolated from both WT and *Adap^–/–^* septic mice were immunostained with antibodies against CD11b and F4/80, followed by flow cytometric analysis. As shown in Figure 3A, 2 distinct subsets of peritoneal cavity cells were identified in both WT and *Adap^–/–^* septic mice: CD11b^+^F4/80^–^ peritoneal cells (P1) and CD11b^+^F4/80^+^ PM subset (P2). Cells within the P1 and P2 clusters from WT and *Adap^–/–^* septic mice were sorted using FACS and subjected to RNA-Seq. Analysis of the RNA-Seq data showed that, while there were only 17 DEGs in CD11b^+^F4/80^–^ peritoneal cells (P1) between WT and *Adap^–/–^* septic mice, 209 DEGs were identified in CD11b^+^F4/80^+^ PMs (P2), including 188 upregulated and 21 downregulated genes, between WT and *Adap^–/–^* mice (Figure 3A). This suggests that changes in gene transcription caused by ADAP deficiency were more profound in the peritoneal cells of the P2 subset than in those of the P1 subset in septic mice. To screen for potential target genes associated with ADAP deficiency–induced aggravation of sepsis, PMs from WT and *Adap^–/–^* mice exposed to LPS in vitro were also subjected to RNA-Seq (Figure 3B). Differential expression analysis revealed a total of 13 shared DEGs between the 2 RNA-Seq datasets of WT versus *Adap^–/–^* PMs isolated from septic mice and WT versus *Adap^–/–^* PMs stimulated with LPS in vitro (Figure 3, B and C); among these, *Pdpn*, a type I mucin-like transmembrane glycoprotein, was consistently identified as one of the most downregulated genes in *Adap^–/–^* PMs compared with WT PMs. To verify the RNA-Seq results, PMs from WT and *Adap^–/–^* mice were subjected to LPS stimulation in vitro, followed by quantitative PCR (qPCR), flow cytometry, and Western blot analysis of PDPN expression. As shown in Figure 3, D–F, PDPN expression at both the mRNA and protein levels was increased in response to LPS stimulation in PMs from WT mice. In sharp contrast, LPS-induced PDPN expression was significantly lower in PMs from *Adap^–/–^* mice. Similar results were also obtained with other macrophages such as RAW264.7 macrophages and immortalized BMMs (iBMMs), wherein loss of ADAP led to a decrease in PDPN expression (Figure 3, G and H). Of note, as shown in Figure 3D, this impairment in LPS-induced PDPN expression did not occur in PMs deficient in Src family-associated phosphoprotein 1 (SKAP1), the de facto ADAP interacting partner (30), wherein LPS stimulated PDPN expression to levels comparable with those in WT macrophages. This suggests that ADAP (but not its binding partner SKAP1) specifically regulates LPS-induced PDPN upregulation in macrophages. This claim was further strengthened by the observation that overexpression of ADAP in ADAP-knockdown RAW264.7 cells could restore LPS-induced PDPN expression (Figure 3I). Moreover, the impairment of LPS-induced PDPN expression persisted in *Adap^–/–^* macrophages cocultured with WT macrophages (Supplemental Figure 1; supplemental material available online with this article; https://doi.org/10.1172/jci.insight.186456DS1), excluding the possibility that differences in cytokine secretion between WT and *Adap^–/–^* PMs caused this impairment. Thus, PDPN is an ADAP-regulated gene in LPS-stimulated macrophages, and LPS-induced PDPN upregulation is ADAP dependent. Together, these data suggest a role for ADAP in TLR4-inducible PDPN upregulation in macrophages.

### The generation of an inducible distinct subset of PDPN^hi^ PMs in vivo is ADAP dependent in a septic mouse model.

**Figure 4:**
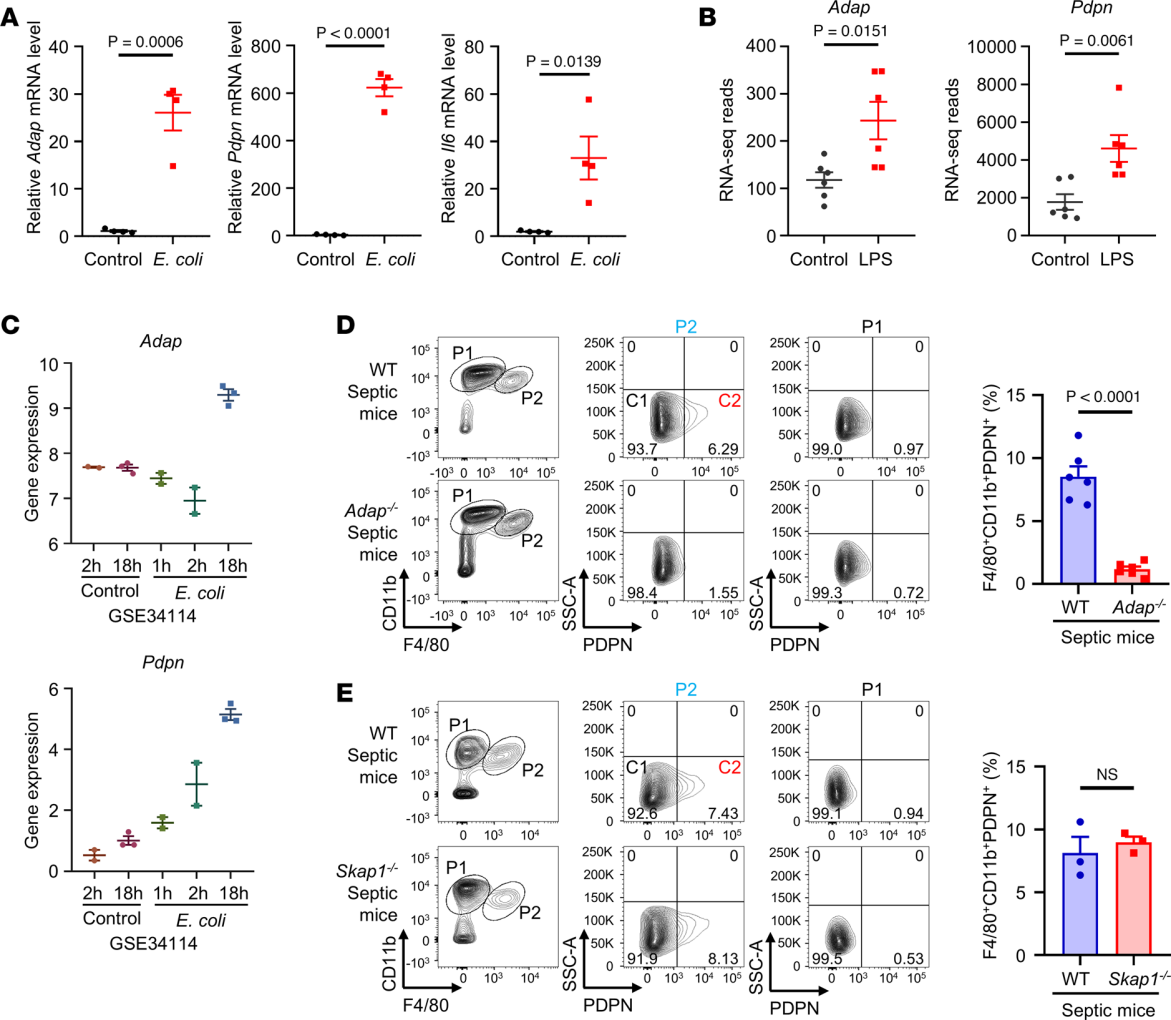
The generation of an inducible distinct subset of PDPN^hi^ PMs in vivo is ADAP dependent in a septic mouse model. (**A**) Vertical scatter plots summarizing the mRNA expression levels of *Adap*, *Pdpn*, and *Il6* as measured by qPCR analysis of peritoneal cells from WT mice 18 hours after injection of saline or *E*. *coli* (2 × 10^7^ CFU, i.p.) (*n* = 4 each, unpaired *t* test). Relative mRNA levels were normalized to *Hprt*. (**B**) Vertical scatter plots showing the expression of *Adap* and *Pdpn* genes (expressed as RNA-Seq reads) extracted from RNA-Seq data of peritoneal cells from LPS-induced peritonitis in mice (*n* = 6 each, unpaired *t* test). (**C**) The expression of *Adap* and *Pdpn* in the peritoneal cells of *E*. *coli*–infected mice was analyzed using a published database (GEO accession no. GSE34114, naive 2 hours, *n* = 2; naive 18 hours, *n* = 3; *E*. *coli* 1 hour, *n* = 2; *E*. *coli* 2 hours, *n* = 2; *E*. *coli* 18 hours, *n* = 3). Gene expression was analyzed in GEO2R. (**D** and **E**) Peritoneal exudate cells were isolated from WT, *Adap^–/–^,* and *Skap1^–/–^* mice 18 hours after injection of *E*. *coli* (2 × 10^7^ CFU, i.p.), and CD11b^+^F4/80^+^PDPN^hi^ macrophages were analyzed by flow cytometry. Left panels: Representative contour plots showing the frequency of PDPN^hi^ macrophages in the peritoneal cavity of WT, *Adap*^–/–^, and *Skap1^–/–^* mice at 18 hours after *E*. *coli* injection. Right panels: Bar graphs showing the percentage of PDPN^hi^ macrophages (**D**, *n* = 6 each; **E**, *n* = 3 each; unpaired *t* test).

We next assessed ADAP-dependent LPS-induced PDPN expression in the PMs in the context of bacterial sepsis. As shown in Figure 4A, the expression of both *Adap* and *Pdpn* was significantly higher in the peritoneal cells of mice with *E*. *coli*–induced sepsis than that in control mice. Furthermore, in line with this, analysis of publicly available transcriptome datasets of septic mouse peritoneal cells (29, 31) revealed an increase in both *Adap* and *Pdpn* expression in peritoneal cells from LPS- and *E*. *coli–*challenged mice (Figure 4, B and C), suggesting a link between ADAP-PDPN expression and bacterial infection during sepsis.

The next question is whether ADAP-dependent LPS-induced PDPN expression can also be applied to generate an inducible subset of PDPN^+^ macrophages in vivo in the context of bacterial sepsis. To address this question, peritoneal cavity cells from septic mice were stained with antibodies against CD11b, F4/80, and PDPN and were subjected to flow cytometry. Indeed, based on their surface PDPN expression levels, there was no induction of PDPN^hi^ PMs in saline-treated WT or *Adap^–/–^* mice (Supplemental Figure 2). However, the CD11b^+^F4/80^+^ PM population (P2) from WT septic mice could be further subdivided into 2 distinct clusters: PDPN^lo^ PMs (C1) and PDPN^hi^ PMs (C2). While the majority (more than 85%) of PMs were C1 that expressed no or very low levels of PDPN, there was a portion (approximately 6%–12%) of CD11b^+^F4/80^+^ PMs that expressed high levels of PDPN (C2) (Figure 4D). Surprisingly, in contrast to WT mice, there was no obvious induction of the C2 subpopulation in *Adap^–/–^* septic mice, wherein the percentage of the induced PDPN^hi^ PM subset was only at marginal levels (Figure 4D), suggesting that PDPN fails to be upregulated in *Adap^–/–^* PMs during sepsis. Interestingly, as a control, induction of the PDPN^hi^ PM subset was also observed in *Skap1^–/–^* septic mice at a level comparable with that in WT septic mice (Figure 4E). We also used CLP, the gold standard in sepsis research, to evaluate responses in polymicrobial sepsis. As shown in Supplemental Figure 3, approximately 20% of the total PMs expressed high levels of PDPN, while no significant induction of the PDPN^hi^ subpopulation was observed in *Adap^–/–^* septic mice. Thus, ADAP deficiency impeded the formation of the PDPN^hi^ PM subpopulation. These data highlight the role of ADAP as a key molecular regulator in the generation of this subpopulation in sepsis.

### The PDPN^hi^ PM subset exhibits a phenotype closely akin to M2 macrophages accompanied by enhanced phagocytic activity, and it provides enhanced protection against sepsis.

**Figure 5:**
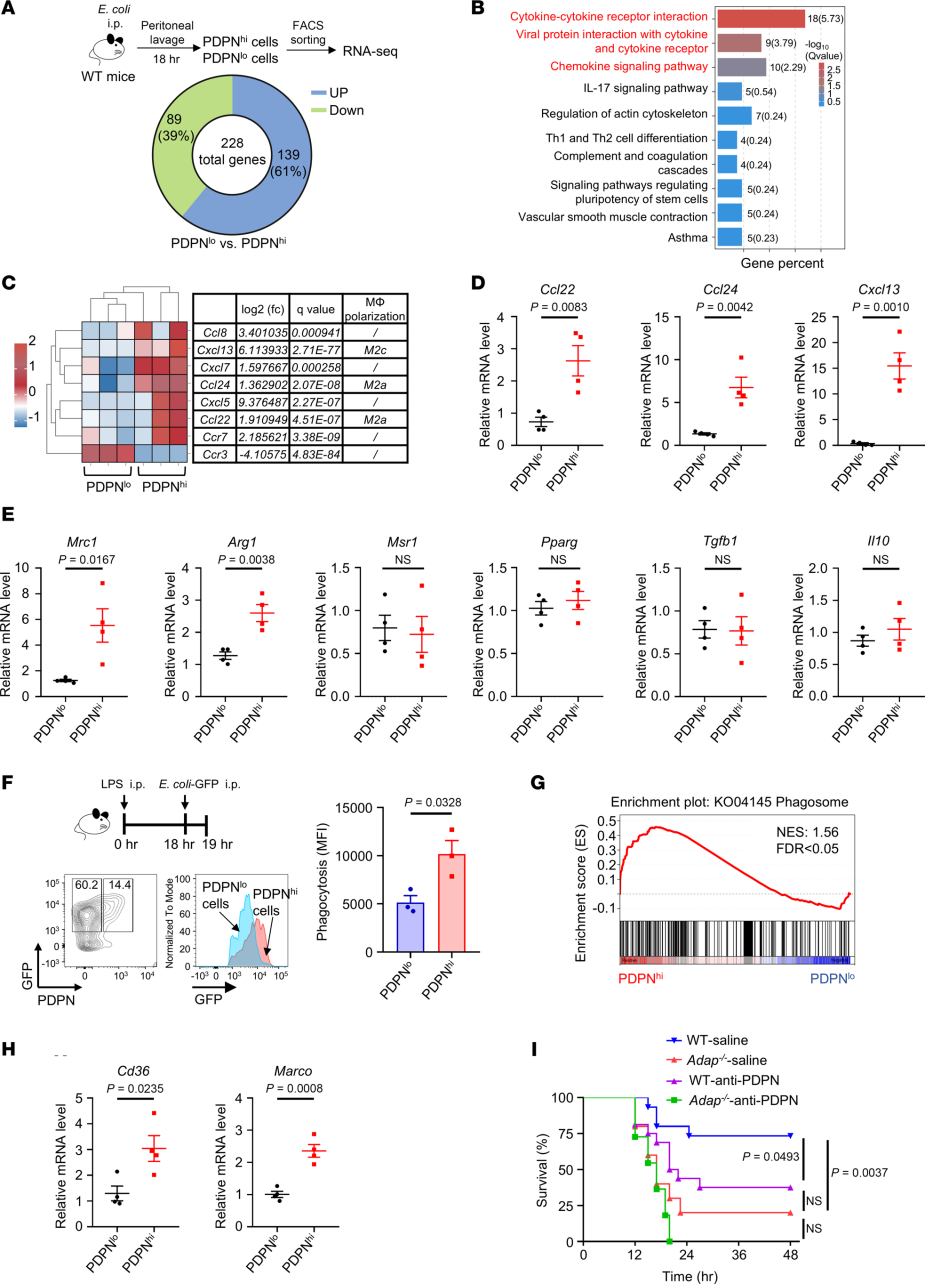
The PDPN^hi^ PM subset exhibits a phenotype closely akin to M2 macrophages accompanied by enhanced phagocytic activity, and provides enhanced protection against sepsis. (**A**) RNA-Seq analysis of CD11b^+^F4/80^+^PDPN^hi^ and CD11b^+^F4/80^+^PDPN^lo^ PMs sorted from WT mice 18 hours after *E*. *coli* injection (2 × 10^7^ CFU, i.p.), showing the numbers of upregulated and downregulated DEGs (log_2_[fold change] ≤ −1/ ≥ 1, 5% FDR). (**B**) Top 10 KEGG pathways identified from DEGs in **A** by KEGG enrichment analysis. (**C**) Heatmaps showing the DEGs of the chemokine-related genes in CD11b^+^F4/80^+^PDPN^hi^ PMs, compared with CD11b^+^F4/80^+^PDPN^lo^, grouped by hierarchical clustering analysis of the RNA-Seq data. (**D** and **E**) qPCR analysis of M2-related chemokine DEGs (**D**) and polarization markers (**E**) in PDPN^hi^ versus PDPN^lo^ PMs from WT septic mice (*n* = 4 each, unpaired *t* test). Relative mRNA levels were normalized to *Hprt*. (**F**) Flow cytometric analysis of *E*. *coli*–GFP uptake by CD11b^+^F4/80^+^PDPN^lo^ and CD11b^+^F4/80^+^PDPN^hi^ PMs in LPS-treated septic WT mice. GFP fluorescence (mean fluorescence intensity) was compared (*n* = 3 each, unpaired *t* test). (**G**) GSEA histogram for the “phagosome” gene set in PDPN^hi^ versus PDPN^lo^ PMs. The normalized enrichment score (NES) and FDR *q* value are indicated. (**H**) The mRNA levels of *Cd36* and *Marco* in PDPN^hi^ and PDPN^lo^ PMs from WT septic mice were determined using qPCR (*n* = 4 each, unpaired *t* test). Relative mRNA levels were normalized to *Hprt*. (**I**) Kaplan-Meier survival analysis of WT and *Adap^–/–^* mice injected with anti-PDPN blocking antibodies (100 μg/mouse, i.v.) 2 hours after *E*. *coli* infection (2 × 10^7^ CFU, i.p.) (WT *E*. *coli*, *n* = 15; *Adap^–/–^* *E*. *coli*, *n* = 10; WT *E*. *coli*–anti-PDPN, *n* = 16; *Adap^–/–^* *E*. *coli*–anti-PDPN, *n* = 11). Log-rank test was used to compare survival curve.

We next set out to figure out the difference in transcriptional profiling between PDPN^hi^ and PDPN^lo^ PM subsets in the context of sepsis. Since no obvious PDPN^hi^ PM subset was induced in *Adap^–/–^* septic mice, we sorted the PDPN^hi^ and PDPN^lo^ PM subsets from WT septic mice infected with *E*. *coli* for 18 hours; these mice were then subjected to RNA-Seq. As shown in Figure 5A, 228 DEGs were identified, with 139 upregulated and 89 downregulated genes between the PDPN^hi^ and PDPN^lo^ PMs. Kyoto Encyclopedia of Genes and Genomes (KEGG) functional analysis revealed that DEGs in PDPN^hi^ PMs compared with PDPN^lo^ PMs were significantly enriched in the cytokine-cytokine receptor interaction pathway and the chemokine signaling pathway (Figure 5B). Notably, upregulation of chemokines, including *Ccl8*, *Cxcl13*, *Cxcl7*, *Ccl24*, *Cxcl5*, and *Ccl22*, was observed in the PDPN^hi^ PMs (Figure 5C), among which CCL22, CCL24, and CXCL13 are associated with macrophage M2 polarization (32). Analysis of macrophage polarization marker gene expression within the RNA-Seq data revealed a greater inclination toward M2-like polarization in PDPN^hi^ PMs (Supplemental Figure 4, A and B). The RNA-Seq results for the elevation in the expression of chemokines *Ccl22*, *Ccl24*, and *Cxcl13* in PDPN^hi^ PMs were further verified using qPCR (Figure 5D). In addition, other key M2 signature genes, including *Mrc1* (*Cd206*) and *Arg-1*, were also significantly elevated in PDPN^hi^ PMs compared with their PDPN^lo^ counterparts (Figure 5E). In contrast, the mRNA expression levels of M1 signature genes such as *Nos2*, *Cd80,* and *Tnf* showed no difference (Supplemental Figure 4C). Moreover, despite the PDPN^hi^ PMs exhibiting elevated expression of M2 macrophage polarization–related chemokines *Ccl22*, *Ccl24*, and *Cxcl13* (Figure 5D), no obvious increase was observed in the expression of the classical antiinflammatory cytokines *Il10* and *Tgfb1* compared with PDPN^lo^ PMs (Figure 5E). Thus, PDPN^hi^ PMs exhibited a phenotype more akin to M2 macrophages, characterized by enhanced expression of CD206, ARG-1, CCL22, CCL24, and CXCL13.

We next sought to assess the functional difference between PDPN^hi^ and PDPN^lo^ PMs in accordance with their phagocytic capacity, which was determined by flow cytometric analysis of the PDPN^hi^ PMs from septic mice infected with living *E*. *coli* expressing GFP. As shown in Figure 5F, the PDPN^hi^ PMs displayed significantly higher phagocytic activity than the PDPN^lo^ PMs, which was consistent with the significant enrichment of the phagosome signaling pathway in PDPN^hi^ PMs relative to that in PDPN^lo^ PMs, as shown by the gene set enrichment analysis (GSEA) of the RNA-Seq data (Figure 5G). Interestingly, in addition to the mannose receptor CD206, the antimicrobial scavenger receptors CD36 and macrophage receptor with collagenous structure (MARCO) were significantly upregulated in PDPN^hi^ PMs compared with PDPN^lo^ PMs (Figure 5H). To further address the function of PDPN^hi^ PMs in host immune defense against bacterial infection in WT and *Adap^–/–^* septic mice, we assessed the effect of blocking sepsis-induced PDPN^hi^ PMs on the severity of sepsis induced by i.p. injection of *E*. *coli*. As previously reported (23, 33), blockade of PDPN^hi^ PMs was achieved via the injection of WT septic mice with anti-PDPN blocking/crosslinking antibody mAb 8.1.1. The PDPN^hi^ PMs blockade resulted in a significant decrease in the survival rate of WT septic mice to a level comparable with that of *Adap^–/–^* septic mice (Figure 5I). Thus, the selective blockade of PDPN^hi^ macrophages exacerbates the severity of sepsis in mice. This suggests a link between the PDPN^hi^ PM subset and ADAP-dependent immune control of bacterial infection in sepsis. Together, ADAP-dependent high expression of PDPN identifies a unique subset of PDPN^hi^ PMs that exert enhanced antibacterial functions.

### BTK-mediated tyrosine phosphorylation of ADAP at Y^571^ is indispensable for TLR4-induced PDPN upregulation in macrophages.

**Figure 6:**
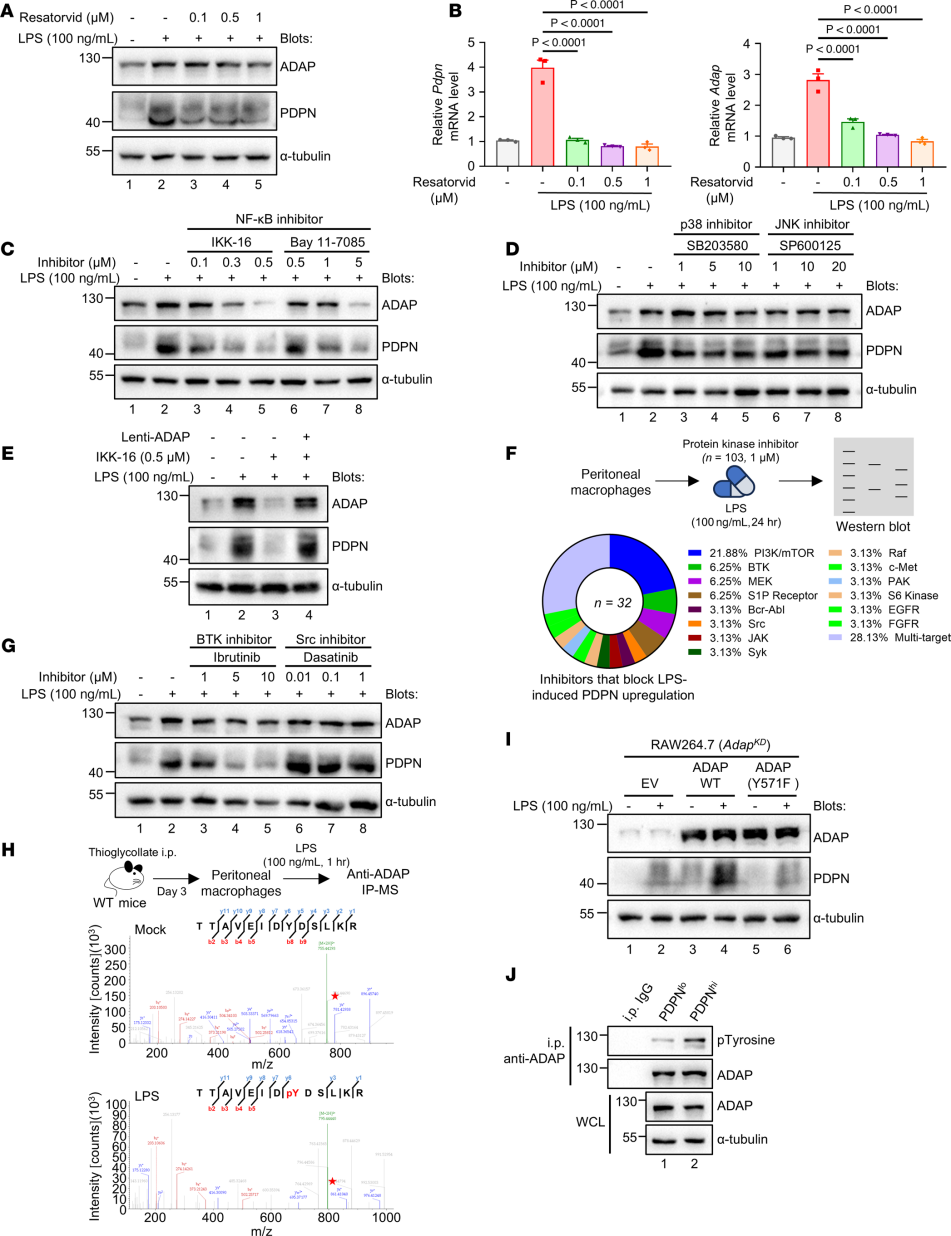
BTK-mediated tyrosine phosphorylation of ADAP at Y^571^ is indispensable for TLR4-induced PDPN upregulation in macrophages. (**A**–**D**) WT PMs were pretreated with resatorvid (0.1, 0.5, and 1 μM), IKK-16 (0.1, 0.3, and 0.5 μM), Bay 11-7085 (0.5, 1, and 5 μM), SB203580 (1, 5, and 10 μM), or SP600125 (1, 10, and 20 μM) for 1 hour, followed by LPS stimulation (100 ng/mL) for 24 hours (**A**, **C**, and **D**) or 12 hours (**B**). ADAP and PDPN expression was analyzed by Western blotting and qPCR (**B**, *n* = 3 each, 1-way ANOVA, Tukey’s multiple-comparison test). Relative mRNA levels were normalized to *Hprt*. α-Tubulin blots are derived from the same samples run contemporaneously in parallel gels (**D**). (**E**) RAW264.7 cells transduced with ADAP were treated with LPS (100 ng/mL, 24 hours) in the presence of IKK-16 (0.5 μM). ADAP and PDPN expression was analyzed by Western blotting. α-Tubulin blots are derived from the same samples run contemporaneously in parallel gels. (**F**) Schematic of kinase inhibitors screening and summary of inhibitors blocking LPS-induced PDPN upregulation. (**G**) WT PMs pretreated with ibrutinib (1, 5, and 10 μM) or dasatinib (0.01, 0.1, and 1 μM) for 1 hour were stimulated with LPS (100 ng/mL, 24 hours). PDPN and ADAP expression was assessed by Western blotting. (**H**) MS confirmed LPS-induced (100 ng/mL, 1 hour) ADAP phosphorylation at Y^571^ in PMs. MS/MS spectra of the phosphorylated peptide (TTAVEIDYDSLKR) are shown. (**I**) ADAP^KD^ RAW264.7 cells reconstituted with empty vector (EV), ADAP-WT, or ADAP (Y571F) were stimulated with LPS (100 ng/mL, 24 hours). PDPN and ADAP expression was analyzed by Western blotting. (**J**) CD11b^+^F4/80^+^PDPN^hi^ (C2) and CD11b^+^F4/80^+^PDPN^lo^ (C1) PMs were sorted from WT septic mice 18 hours after *E*. *coli* injection (2 × 10^7^ CFU, i.p.). Lysates were immunoprecipitated with anti-ADAP and immunoblotted for p-Tyr^100^ and ADAP.

Given that LPS-induced PDPN expression in macrophages to form the PDPN^hi^ PM subpopulation in sepsis is dependent on ADAP, we next sought to elucidate the signaling pathways involved in this process. TLR4 is a pattern recognition receptor of macrophages that acts as a sensor of LPS (34). As shown in Figure 6, A and B, the induction of both ADAP and PDPN by LPS in macrophages was inhibited by the TLR4 inhibitor resatorvid, indicating that the upregulation of ADAP and PDPN in macrophages relies on TLR4-mediated signal transduction. TLR4 stimulation activates classical downstream signaling pathways such as the NF-κB signaling pathway and MAP kinases (p38, JNK, and ERK) (35). To confirm this observation, pharmacological inhibitors of IκB kinase (IKK-16 and Bay 11-7085) reduced the expression of both ADAP and PDPN in TLR4-stimulated macrophages (Figure 6C), whereas the inhibitors of p38 (SB203580) and JNK (SP600125) had only marginal effects (Figure 6D). Furthermore, the overexpression of ADAP in RAW264.7 cells counteracted the inhibitory effects of NF-κB inhibitors on PDPN expression (Figure 6E), suggesting that the NF-κB–dependent induction of ADAP may participate in signaling pathways other than the classical NF-κB pathway to regulate LPS-induced PDPN expression in macrophages.

Protein kinases are critical components of TLR4-mediated signal transduction (36). To further identify the kinases downstream of TLR4 signaling associated with ADAP-dependent PDPN induction following LPS stimulation in macrophages, we screened a kinase inhibitor library consisting of 103 inhibitors targeting different kinases to identify kinase inhibitors that can prevent LPS-induced upregulation of PDPN in macrophages. As shown in Figure 6F, Supplemental Figure 5, and Supplemental Table 1, a total of 32 compounds, including 2 BTK inhibitors and 7 PI3K/mTOR inhibitors, blocked LPS-induced PDPN expression in macrophages. Consistently, our previous study showed that LPS stimulation induces tyrosine phosphorylation at Y^571^ in the YDSL motif of ADAP by BTK (14). Therefore, we first analyzed the effect of BTK inhibition on LPS-induced PDPN expression in macrophages. As shown in Figure 6G, LPS-induced PDPN expression in macrophages was decreased upon treatment with the BTK inhibitor ibrutinib but not with the Src inhibitor dasatinib, even though Src kinase interacts with ADAP at Y^807^ to regulate osteoclast migration and progression (37). This suggests that BTK is one of the major kinases specifically responsible for ADAP-dependent PDPN induction by LPS stimulation in macrophages. Furthermore, we assessed the significance of ADAP phosphorylation at Y^571^ in the upregulation of PDPN expression in macrophages in response to LPS stimulation. As shown in Figure 6H, indeed, mass spectrometry (MS) analysis verified that tyrosine phosphorylation of ADAP at Y^571^ was the sole modification observed in the PMs following LPS stimulation. Importantly, reintroduction of WT ADAP successfully restored the impaired induction of PDPN by LPS in ADAP-knockdown RAW264.7 cells, whereas reconstitution of the ADAP Y571F mutant had only a minor effect, comparable with that of the empty vector (EV) (Figure 6I), indicating that tyrosine phosphorylation of ADAP at Y^571^ is critical for the LPS-induced upregulation of PDPN in macrophages. Interestingly, PDPN^hi^ PMs from septic mice exhibited enhanced tyrosine phosphorylation of ADAP compared with their PDPN^lo^ counterparts (Figure 6J), suggesting that the phosphorylation status of ADAP serves as a molecular mechanism controlling TLR4-induced PDPN upregulation in PMs. Together, these data demonstrate that phosphorylation of ADAP at Y^571^, mediated by BTK, is essential for sustaining the induction of PDPN in macrophages in response to LPS or in the context of sepsis.

### mTOR-mediated STAT3 phosphorylation potentiates ADAP-STAT3–dependent PDPN transcription in response to TLR4 stimulation.

**Figure 7:**
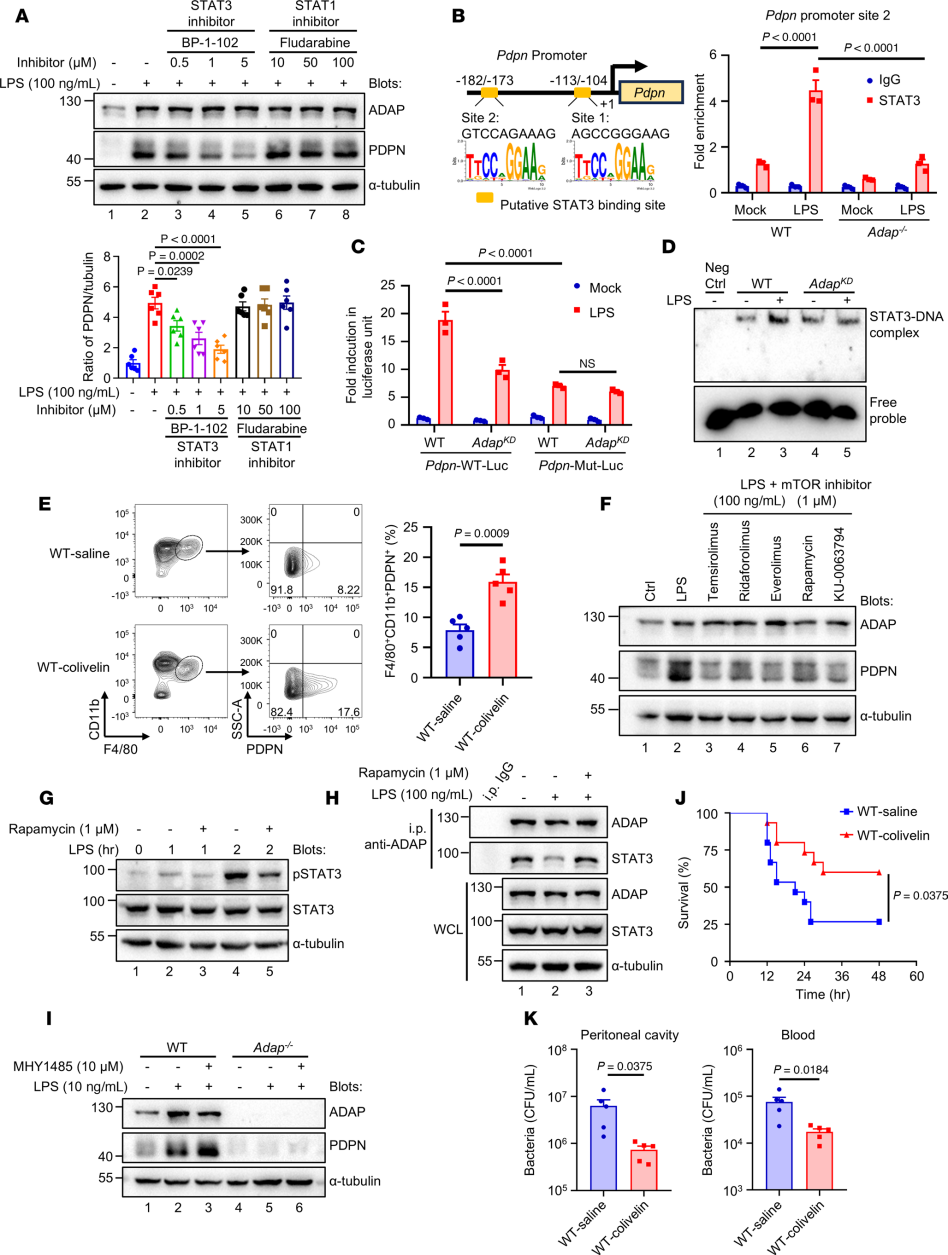
mTOR-mediated STAT3 phosphorylation potentiates ADAP-STAT3–dependent *PDPN* transcription in response to TLR4 stimulation. (**A**) PDPN expression in PMs pretreated with BP-1-102 (0.5, 1, and 5 μM) or fludarabine (10, 50, and 100 μM) for 1 hour and stimulated with LPS (100 ng/mL, 24 hours), analyzed by Western blotting and densitometry normalized to α-tubulin (*n* = 6 each, 1-way ANOVA, Tukey’s multiple-comparison test). (**B**) STAT3 binding motifs in the *Pdpn* promoter and CUT & RUN qPCR showing STAT3 enrichment at site 2 in untreated or LPS-treated PMs (100 ng/mL, 1 hour) (*n* = 3 each, 2-way ANOVA, Tukey’s multiple-comparison test). (**C**) Luciferase activity of *Pdpn*-WT-Luc or *Pdpn*-Mut-Luc in WT or ADAP^KD^ RAW264.7 cells following LPS stimulation (100 ng/mL, 6 hours) (*n* = 3 each, 2-way ANOVA, Tukey’s multiple-comparison test). (**D**) EMSA showing STAT3-DNA binding in nuclear extracts of WT or ADAP^KD^ RAW264.7 cells after LPS stimulation (100 ng/mL, 1 hour). (**E**) Flow cytometry of CD11b^+^F4/80^+^PDPN^hi^ PMs in WT mice treated with colivelin (1 mg/kg, i.p.) after *E*. *coli* infection (2 × 10^7^ CFU, i.p.) (*n* = 5 each, unpaired *t* test). (**F** and **G**) Western blot of PDPN, p-STAT3, and STAT3 in LPS-treated PMs (100 ng/mL) with or without mTOR inhibitors or rapamycin (1 μM). (**H**) LPS-stimulated PMs (100 ng/mL, 1 hour) pretreated with rapamycin (1 μM, 1 hour) were immunoprecipitated with anti-ADAP and immunoblotted for STAT3. (**I**) PDPN expression in WT and *Adap^–/–^* PMs treated with LPS (10 ng/mL) and MHY1485 (10 μM, 24 hours) was analyzed by Western blotting. (**J** and **K**) Kaplan-Meier survival curves and bacterial burden in WT mice treated with colivelin (1 mg/kg, i.p.) after *E*. *coli* infection (5 × 10^7^ CFU, i.p.), showing survival (*n* = 15 each, log-rank test) and bacterial load in peritoneal lavage fluid and blood at 18 hours (*n* = 5 each; unpaired *t* test).

Our previous study showed that ADAP interacts with STAT3 and that ADAP phosphorylation at Y^571^ is essential to prime STAT3 for activation (14). This prompted us to investigate whether STAT3 is an effector downstream of TLR4-BTK-ADAP signaling axis that directly regulates PDPN transcription in macrophages. As shown in Figure 7A, treatment with the STAT3 inhibitor BP-1-102 significantly inhibited LPS-induced PDPN expression in macrophages, in contrast to the marginal effect of the STAT1 inhibitor fludarabine. This suggests that STAT3, but not STAT1, specifically regulates LPS-induced PDPN expression in macrophages. Next, to assess whether STAT3 can directly transactivate the *Pdpn* gene, prediction of transcription factor-binding sites in the promoter regions of *Pdpn* using the JASPAR database identified 2 putative STAT3 binding sites: –113 (5′-AGCCGGGAAG-3′) –104 (site 1) and –182 (5′-GTCCAGAAAG-3′) –173 (site 2) (Figure 7B). To verify that these putative STAT3 binding sites are functional in the *Pdpn* promoter, CUT & RUN qPCR analyses were performed, showing that LPS could effectively stimulate STAT3 binding predominantly to site 2 rather than site 1 of the *Pdpn* promoter in macrophages (Figure 7B and Supplemental Figure 6). LPS-induced STAT3 binding to site 2 of the *Pdpn* promoter was substantially lower in macrophages lacking ADAP (Figure 7B). Furthermore, a luciferase reporter assay showed that either mutation of STAT3 binding site 2 or knockdown of ADAP expression in RAW264.7 cells decreased *Pdpn* promoter-driven luciferase activity following LPS stimulation (Figure 7C). Moreover, the gel shift assay showed that STAT3 formed distinctive supershifted complexes with the oligonucleotide probe containing STAT3 binding site 2 of the *Pdpn* promoter, which was enhanced upon LPS stimulation (Figure 7D). This complex formation was reduced in the nuclear extract of ADAP-knockdown RAW264.7 cells (Figure 7D). These data demonstrate that ADAP-dependent induction of PDPN in macrophages in response to LPS stimulation is mainly mediated by STAT3, which regulates *Pdpn* transcription by binding to site 2 of the *Pdpn* promoter. Moreover, we examined the effects of STAT3 activation on the generation of PDPN^hi^ PMs in septic mice in vivo. Colivelin, a STAT3 agonist, has been reported to have therapeutic potential for the treatment of sepsis-associated endothelial dysfunction (38). As shown in Figure 7E, the injection of WT mice with colivelin significantly increased the percentage of the induced PDPN^hi^ PM subset from 8.2% to 17.6% in WT septic mice, demonstrating a correlation between STAT3 activation and the enhanced induction of PDPN expression in PMs.

Since ADAP lacks intrinsic kinase activity, its influence on STAT3 activation should be indirect through other intracellular protein kinases that could directly activate STAT3 via phosphorylation. Thus, it is necessary to identify the kinases responsible for STAT3 activation. Our kinase inhibitor library screening experiment revealed that in addition to BTK inhibitors, 7 of 32 molecules (about 21.88%) belonging to the class of PI3K/mTOR inhibitors could also suppress LPS-induced PDPN expression in macrophages (Figure 6F), some of which, such as rapamycin, exhibited inhibitory effects on LPS-induced PDPN expression in vitro, as verified by Western blot analysis (Figure 7F). Interestingly, in TLR4-stimulated macrophages, the mTOR inhibitor rapamycin treatment led to a decrease in LPS-induced STAT3 phosphorylation at Y^705^ (Figure 7G, lane 5 versus lane 4), which is a determinant of STAT3 nuclear translocation and DNA binding (39). These data indicate that mTOR may activate STAT3 during LPS-induced transactivation of *PDPN* transcription in macrophages. Moreover, treatment with the mTOR inhibitor rapamycin prevented LPS-induced dissociation of the ADAP-STAT3 complex, which marks STAT3 activation (Figure 7H, lane 3 versus lane 2) (14). Conversely, treatment with the mTOR agonist MHY1485 resulted in an increase in LPS-induced PDPN expression in PMs from WT mice but not in PMs from *Adap^–/–^* mice (Figure 7I). Together, these findings demonstrate that LPS-induced *PDPN* transcription is directly regulated by STAT3 in macrophages, which is coregulated by both ADAP phosphorylation at Y^571^ mediated by BTK and STAT3 phosphorylation at Y^705^ by mTOR downstream of TLR4 stimulation.

Given that the STAT3 agonist colivelin could increase the percentage of the induced PDPN^hi^ PM subset in WT septic mice, we sought to determine its therapeutic potential for the treatment of sepsis. As shown in Figure 7J, the mortality rate of high-dose *E*. *coli*–induced (5 × 10^7^ CFU/mouse) septic mice was reduced after treatment with colivelin compared with that of saline-treated WT mice. Moreover, colivelin treatment significantly reduced the bacterial burden in both the peritoneal lavage fluid and blood from WT septic mice (Figure 7K). Thus, these findings demonstrate that STAT3 signaling axis activation to maintain PDPN^hi^ PMs formation is a promising therapeutic target for the treatment of sepsis.

## Discussion

Sepsis-induced immune regulation is associated with macrophage reprogramming (40). Type I mucin-like transmembrane protein PDPN, the only known endogenous ligand for CLEC-2, is induced in macrophages in response to LPS stimulation or bacterial infection during sepsis (17, 23, 24). However, the molecular mechanisms underlying the precise regulation of TLR4-dependent induction of PDPN in macrophages and the role of the PDPN^hi^ macrophage subpopulation in sepsis immunity have not yet been elucidated. In this study, we demonstrated that ADAP is indispensable for the induction of PDPN in TLR4-stimulated macrophages to reset macrophage function in vitro and in vivo. ADAP deficiency is associated with an increased severity of sepsis. The TLR4-induced PDPN^hi^ PM subpopulation is endowed with enhanced phagocytic activity, is akin to an M2 phenotype, and plays a direct role in protecting against bacterial infection in sepsis. This is in contrast to existing models of the indirect effect of macrophage PDPN in sepsis, in which PDPN on macrophages regulates the inflammatory reaction via interaction with platelet CLEC-2 (23).

In addition to PDPN, ADAP is elevated in macrophages in response to LPS or bacterial challenge during sepsis, suggesting that PDPN and ADAP play related roles in modulating macrophage function during sepsis. Indeed, LPS-induced PDPN expression in macrophages at both mRNA and protein levels was dependent on ADAP in vitro and in vivo. This is a paradigm for ADAP, an immune adaptor, in the regulation of surface molecule expression on macrophages during sepsis. Interestingly, other examples of ADAP regulating the surface expression of membrane molecules on immune cells include promoting PD-1 surface expression in cytotoxic T lymphocytes via the transcription factor NFATc1 during antitumor immunity (41) and inhibiting FcγRI/IV surface expression on macrophages via STAT1 in immune thrombocytopenia (15). NK cells lacking ADAP are characterized by reduced CD107a surface expression during infection with the intracellular pathogen Listeria monocytogenes (42). This evidence suggests that, despite being an immune adaptor, ADAP has a propensity to regulate various immune cell functions by modulating the surface expression of certain functional molecules.

It was also the case in vivo that PDPN expression was induced in macrophages in septic mice in an ADAP-dependent manner. A portion of PMs expressing high levels of PDPN (PDPN^hi^ PMs) was identified in WT and *Skap1^–/–^* septic mice but not in *Adap^–/–^* septic mice (Figure 4, D and E). Compared with the PDPN^lo^ PM counterpart, this induced PDPN^hi^ subpopulation exhibited enhanced phagocytic ability and was closely akin to an M2-polarized phenotype, characterized by upregulation of the M2-related signature markers CD206 and ARG-1 and chemokines such as CCL22, CCL24, and CXCL13 (Figure 5). This is consistent with the observation that PDPN^hi^ tumor–associated macrophages exhibit the M2 phenotype (26) and that tumor-derived PDPN–containing extracellular vehicles mediate M2-like polarization of macrophages (43). These results are also in line with the notion that M2 macrophages generally have higher phagocytic activity than M1 macrophages and have a protective role against microbial infection (44). Moreover, blockade of the PDPN^hi^ PM subset by administration of an anti-PDPN antibody aggravates disease severity, corroborating the protective role of this unique PDPN^hi^ PM subpopulation in host defense against sepsis. This is consistent with previous findings that blockade of PDPN leads to an increased cytokine storm and bacterial proliferation in mice with peritonitis (23). It is not surprising that ADAP deficiency aggravated sepsis in a mouse septic model, and septic nonsurvivors had markedly decreased *ADAP* mRNA levels compared with septic survivors (Figure 1C). Thus, these data illustrate that PDPN marks a distinct M2-like PDPN^hi^ PM subpopulation with enhanced phagocytic ability, which responds differentially to TLR4 signaling compared with its PDPN^lo^ counterparts.

Given that LPS-induced PDPN is ADAP dependent, unexpectedly, the level of *ADAP* expression was not significantly higher in the PDPN^hi^ PMs subpopulation than in its PDPN^lo^ counterparts (Supplemental Figure 7). What differed was the level of ADAP tyrosine phosphorylation, which was higher in the PDPN^hi^ PM subpopulation compared with the PDPN^lo^ counterpart (Figure 6J), suggesting that the activation status of ADAP is crucial for LPS-induced PDPN expression in macrophages. Our unbiased screening of a protein kinase inhibitor library identified 2 BTK inhibitors and 7 PI3K/mTOR inhibitors that could block LPS-induced upregulation of PDPN in TLR4-stimulated macrophages in vitro. This was supported by the observation that LPS-induced upregulation of PDPN could be efficiently suppressed by treating macrophages with the BTK inhibitor ibrutinib, which inhibited ADAP Y^571^ tyrosine phosphorylation without affecting ADAP expression in TLR4-stimulated macrophages (Figure 6G). Further evidence came from the impairment of LPS-induced PDPN expression in ADAP-knockdown macrophages, which was largely restored by lentiviral-mediated reconstitution of ADAP but not by the ADAP Y571F mutant (Figure 6I).

**Figure 8:**
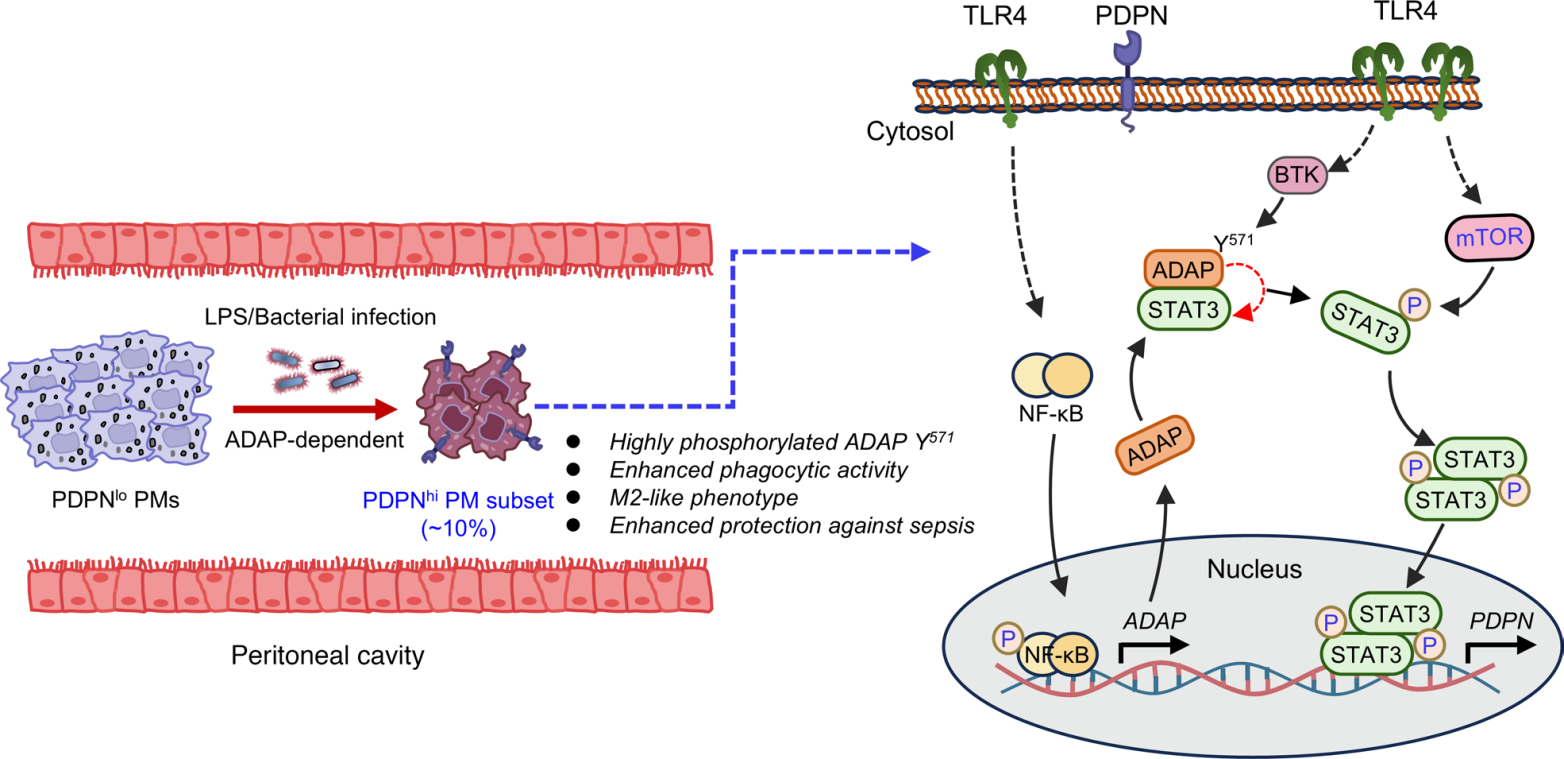
Schematic model for the functional role of ADAP in controlling LPS-induced PDPN expression to form the PDPN^hi^ PM subset during sepsis. ADAP is upregulated in sepsis and serves as an LPS stimulus–responsive protein that governs the upregulation of PDPN to form a distinct PDPN^hi^ PM subset during sepsis, which displays a phenotype closely akin to M2 macrophages with enhanced phagocytic activity and provides enhanced protection against sepsis in the host. Mechanistically, while TLR4 signaling stimulates the upregulation of ADAP via the NF-κB pathway, TLR4-triggered phosphorylation of ADAP at Y^571^ by BTK primes the subsequent direct phosphorylation of STAT3 by mTOR, which transactivates *PDPN* transcription.

In summary, we propose a model for the ADAP-dependent LPS-induced surge of PDPN to form a PDPN^hi^ PM subset as a critical response of macrophages to bacterial infection (Figure 8). Mechanistically, ADAP-dependent TLR4-activated STAT3 phosphorylation for *PDPN* transcription involves 2 processing steps: (a) initial TLR4 stimulated tyrosine phosphorylation of ADAP at Y^571^ by BTK and (b) subsequent STAT3 phosphorylation mediated by mTOR. During this process, tyrosine phosphorylation of ADAP at Y^571^ by BTK facilitates STAT3 phosphorylation by mTOR, causing STAT3 to dissociate from ADAP. Thus, these 2 events converge with STAT3 activation in the transactivation of *PDPN* transcription in TLR4-stimulated macrophages. In support of this, treatment with a STAT3 agonist significantly increased the percentage of the PDPN^hi^ PM subset in vivo (Figure 7E). Furthermore, the STAT3 agonist colivelin could improve the survival rate and reduce the bacterial burden in WT septic mice (Figure 7, J and K). When ADAP is underexpressed, LPS fails to induce PDPN expression in macrophages, resulting in a decrease in the generation of PDPN^hi^ PMs and in aggravation of sepsis. This model indicates that effectively and specifically activating the BTK-ADAP–dependent mTOR-STAT3 signaling axis to maintain the formation of PDPN^hi^ PMs may represent a promising target for immunotherapies in inflammatory diseases such as sepsis.

## Methods

### Sex as a biological variable.

Sex was not considered as a biological variable. Both male and female mice were included in this study.

### Antibodies and reagents.

The antibodies used in this study are listed as follows: rabbit anti-ADAP (MilliporeSigma, 07-546), rabbit anti–α-tubulin (Abcam, ab4074), rat anti-PDPN (Abcam, ab256559), rabbit anti-Stat3 (Proteintech, 10253-2-AP), rabbit anti–p-Stat3 (tyr705) (CST, 9131), mouse anti–p-tyrosine (p-Tyr^100^) (CST, 9411), HRP-conjugated goat anti-rat IgG (H+L) (ABclonal, AS028), HRP-conjugated goat anti–rabbit IgG (H+L) (CST, 7074), PE-Cy7–conjugated anti–mouse CD45 (BD Bioscience, 552848), FITC-conjugated anti–mouse CD11b (BD Bioscience, 553310), Alexa Fluor 647–conjugated anti–mouse F4/80 (BD Bioscience, 565853), PE anti–mouse PDPN (BD Bioscience, 566390), PE-conjugated anti–mouse Ly-6G (BioLegend, 127608), Alexa Fluor 647–conjugated anti–mouse PDPN (BioLegend, 156204), InVivoMAb polyclonal Syrian hamster IgG (BioXCell, BE0087), and InVivoMAb anti–mouse PDPN (gp38) (BioXCell, BE0236).

LPS derived from *E. coli* strain O111:B4 (catalog L4391) and 055:B5 (catalog L6529) were obtained from Sigma-Aldrich. Clodronate liposomes (catalog 40337ES08) and PBS liposomes (catalog 40338ES08) were obtained from Yeasen. Colivelin (catalog TP1856), BP-1-102 (catalog T3708), and MHY1485 (catalog T1908) were obtained from Topscience. Resatorvid (catalog HY-11109) was obtained from MedChemExpress. Fludarabine monophosphate (catalog F0913) was obtained from TCI Chemicals. Kinase inhibitor library (catalog L1200), IKK-16 (catalog S2882), Bay 11-7085 (catalog S7352), SB203580 (catalog S1076), SP600125 (catalog S1460), Temsirolimus (catalog S1044), Ridaforolimus (catalog S1022), Everolimus (catalog S1120), Rapamycin (catalog S1039), KU-0063794 (catalog S1226), Ibrutinib (catalog S2680), and Dasatinib (catalog S1021) were obtained from Selleck Chemicals.

### Clinical specimens.

Patients diagnosed with sepsis according to the guidelines of The Third International Consensus Definitions (Sepsis-3) and age-matched healthy controls with no history or clinical disease were enrolled (45). Blood samples from patients with sepsis were collected at the time of intensive care unit (ICU) admission. The healthy controls were from volunteers.

### Animals.

*Adap*^–/–^ and *Skap1*^–/–^ mice were gifts from C.E. Rudd (University of Cambridge, Cambridge, United Kingdom) and were originally generated by Peterson et al. (46) and Wang et al. (30), respectively. WT C57BL/6J mice were obtained from GemPharmatech. All mice were housed in specific pathogen–free facilities at Soochow University. Age- and sex-matched mice between 6 and 8 weeks were used.

### Primary cells and cell lines.

PMs and iBMDMs were prepared as previously described (14). PBMC was isolated from blood samples using standardized density gradient technique (Ficoll-Paque, GE Healthcare, 17-1440-03). Primary human macrophages were generated by enriching monocytes from PBMCs by adherence to a culture plate for 2 hours, followed by washing with PBS to remove nonadherent cells, and culturing the adherent monocytes in RPMI 1640 complete medium with 100 ng/mL M-CSF (Sino Biological, 11792-HNAH) for 7 days. RAW264.7 and THP-1 cell lines were purchased from the American Type Culture Collection (ATCC). RAW264.7 cells were maintained in DMEM supplemented with 10% FBS and 100 U/mL penicillin/streptomycin. THP-1 cell line was cultured in RPMI 1640 complete medium.

### Sepsis model.

For the sepsis model, mice were injected i.p. with 200 μL of a suspension of live *E*. *coli* (ATCC 25922; 2 × 10^7^ CFU/mouse). After 18 hours, macrophage count in the peritoneal lavage fluid was measured by flow cytometry using anti-CD45, -CD11b, and -F4/80, and neutrophils were analyzed using anti-Ly6G antibody. For PDPN neutralization, mice were i.v. injected with anti-PDPN blocking antibodies (BioXCell, BE0236) (100 μg/mouse) or isotype control (BioXCell, BE0087) 2 hours after *E*. *coli* injection. For STAT3 activation in vivo, a single dose of the STAT3 agonist colivelin (1 mg/kg weight) was administered i.p. 1 hour after infection with *E*. *coli*. CLP surgery was performed as described previously (47). Briefly, a midline laparotomy was performed, the cecum was exteriorized, and 20% of the cecum was ligated and punctured with a 21G needle, followed by extrusion of a small drop of fecal contents. The cecum was then returned to the peritoneal cavity, and the incision was closed using 2 layers of sutures. Saline (1 mL) was injected s.c. for resuscitation immediately after closing the abdomen.

### Bacterial burden assay.

Peritoneal lavage fluid or blood was diluted serially in sterile PBS. Each dilution (25 μL of each) was aseptically plated on LB agar at 37°C. After 14–18 hours of incubation, bacterial colonies were counted and expressed as CFU/mL of blood or peritoneal lavage fluid.

### Histological analysis.

For organ pathology analysis, the mice were sacrificed 18 hours after *E*. *coli* injection. Lung tissues were fixed in 4% paraformaldehyde for 24 hours and embedded in paraffin. After removal of paraffin, the tissues were sectioned and stained using the standard H&E procedure. A blinded observer was assigned to evaluate the histopathological pulmonary injury. The severity of lung injury was scored as described previously (48). Briefly, lung injury was scored based on the degrees of edema, hemorrhage, inflammatory cell infiltration, and histopathological changes, with 0: absent of injury; 1: modest injury; 2: intermediate injury; 3: widespread injury; and 4: severe injury.

### Phagocytosis assay.

WT mice were injected with LPS at a dose of 10 mg/kg, and after 18 hours of LPS treatment, the mice were challenged with *E*. *coli*–GFP (2 × 10^7^ CFU, i.p.) for 1 hour. Cells from the peritoneal lavage fluid were collected, washed with cold PBS 3 times, and prepared for flow cytometric analysis. The mean fluorescence intensity (MFI) was calculated using FlowJo software (Tree Star Inc.) and was used as a measure of phagocytosis.

### Macrophage depletion and reconstitution.

Liposome-based macrophage depletion followed by BMDM reconstitution was described previously (49). Liposome-encapsulated clodronate (5 mg/mL) was administered i.v. to deplete macrophages at a dose of 10 mL/kg, 3 days before *E*. *coli* challenge. Macrophage adoption transfer was performed by i.v. injection of 1 × 10^6^ BMDMs 2 days after macrophage depletion.

### qPCR.

Total RNA was extracted from tissues or cells using TRIzol Reagent (Sigma-Aldrich, T9424), according to the manufacturer’s instructions. First-strand cDNA was prepared from RNA using the Hifair II 1st Strand cDNA Synthesis Kit (Yeasen, 11119ES60). qPCR was carried out using the Hieff Quantitative PCR SYBR Green Master Mix (Yeasen, 11184ES03) on the QuantStudio Design and Analysis System (Applied Biosystems). The relative expression levels were calculated using the standard ΔΔCt method and normalized to the housekeeping gene. Primers were synthesized by Sangon Biotech, and the primer sequences are listed in Supplemental Table 2.

### Flow cytometry.

A single-cell suspension was prepared and washed with FACS buffer (2% FCS and 2 mM EDTA in PBS). The cells were stained with antibodies for 30 minutes and fixed in 2% paraformaldehyde. Data were acquired on a FACSCanto II flow cytometer and analyzed using FlowJo software.

### Plasmids, lentiviral transduction, and luciferase reporter assay.

ADAP knockdown and overexpression of RAW264.7 cells were established by lentivirus infection as described previously (15). Briefly, cDNA encoding full-length mouse *Adap* (NCBI Gene ID: 23880) or shRNAs were packaged with psPAX2 (Addgene, 12260) and pMD2.G (Addgene, 12259) into HEK293T cells and were delivered to the target cells by lentiviral transduction in the presence of 8 μg/mL polybrene. Cells were selected with puromycin for at least 3 weeks before experimental use. *Pdpn*-LUC was constructed by inserting fragments from the *Pdpn* promoter (from –1,000 to +1) into the pTA-LUC plasmid. For the luciferase reporter assay, 5 × 10^5^ RAW264.7 cells were transfected with 5 μg of *Pdpn*-Luc and 0.5 μg of pRL-TK Renilla luciferase control plasmid (Promega) for 24 hours, followed by treatment with 100 ng/mL LPS for 6 hours. Cells were lysed, and firefly luciferase activity was measured and normalized to *Renilla* luciferase activity following the manufacturer’s instructions (Promega).

### Immunoblotting and immunoprecipitation.

Cell lysis, immunoprecipitation, and detection were performed as described previously (14). Briefly, Cells were lysed in lysis buffer (1% Triton X-100 [v/v] in 20 mM Tris-HCl [pH 8.3] and 150 mM NaCl) supplemented with an EDTA-free protease inhibitor cocktail (Roche). For immunoprecipitation experiments, cell lysates were precleared with protein G Sepharose beads (GE Healthcare, 17061801), followed by incubation with antibodies at 4°C overnight. Immunoprecipitates were resolved by SDS-PAGE, and immunoblotting was performed using the indicated primary antibodies. Immunoblots were developed with HRP-conjugated secondary antibodies and visualized by enhanced chemiluminescence (ECL) reagents (Yeasen, 36208ES76).

### Cleavage under targets and release using nuclease assay.

CUT & RUN assays were performed using the Hyperactive pG-MNase CUT & RUN Assay Kit for PCR/qPCR (Vazyme, HD101), according to the manufacturer’s instructions. Briefly, 1 × 10^6^ PMs isolated from WT or *Adap^–/–^* mice were stimulated with LPS (100 ng/mL) for 1 hour. The cells were collected and incubated with ConA beads for 10 minutes at room temperature. The cell-beads complex was incubated with IgG control and STAT3 antibodies (Proteintech, 10253-2-AP) at 4°C overnight. After binding to the pG-MNase enzyme, the cell-beads complex was fragmented using CaCl_2_ solution at 4°C for 2 hours. Stop buffer was added to stop fragmentation, and DNA was extracted using column-based extraction reagents. The resulting DNA products were quantified by qPCR, and spike-in DNA was used as a control. The sequences of the primers for the *Pdpn* promoter were as follows: site 1, sense 5′-GCTGAGACTTTTGCTCAGCG-3′, anti-sense 5′-CTTGATCTCGTTGGAGCCTCA-3′; site 2, sense 5′-CCGGGACCGGAGACATAAAT-3′, anti-sense 5′-GAGCAAAAGTCTCAGCGCCA-3′.

### Electrophoresis mobility shift assay (EMSA).

A nuclear protein extraction kit (Yeasen, 20126ES50) and a chemiluminescent EMSA kit (Beyotime, GS009) were used in accordance with the manufacturer’s protocol. Nuclear extracts from untreated or LPS-treated (100 ng/mL, 1 hour) WT and ADAP-knockdown RAW264.7 cells were incubated with biotin-labeled probes designed to target putative binding sites in the *Pdpn* promoter. Two double-stranded oligonucleotide probes (site 2: 5′-CTGCGAGTCCAGAAAGCCCGGGCAC-3′) were 5′ end-labeled with biotin. A probe lacking the nuclear extract was used as a negative control, and the biotin-labeled protein/DNA complex was visualized with streptavidin-HRP.

### Liquid chromatography–MS/MS analysis.

The phosphorylation sites in LPS-stimulated ADAP were identified as described previously with minor modifications (14). Briefly, PMs were harvested, stimulated with LPS (100 ng/mL) for 1 hour, and then lysed. Total ADAP was pulled down by immunoprecipitation, as described above. Proteins of interest were dissociated from the beads using 8M urea, followed by reduction, alkylation, and digestion according to standard protocols (50). Tryptic extracts were collected, lyophilized, resuspended in 0.1% formic acid, and tested on Linear ion trap Orbitrap combined mass spectrometer (LTQ Orbitrap Elite ETD) (Thermo Fisher Scientific) for liquid chromatography–MS/MS (LC-MS/MS) analysis.

### Bioinformatics analysis.

Gene expression datasets were obtained from the GEO database of the National Center for Biotechnology Information. Expression data were analyzed using the GEO2R online tool. The RNA-Seq data of the peritoneal tissue from mice with LPS-induced peritonitis were obtained from the supplementary file of the study by Liu et al. (31). A Venn diagram was generated to obtain overlapping DEGs in the 4 datasets (GSE1871, GSE17355, GSE165226, and GSE2411) generated using murine lungs in response to LPS or cecal ligation and puncture stimulation. Data on genomic expression were screened using the online tool GEO2R package limma to identify DEGs, which were defined as fold change ≥ 2 and adjuseted (adj.) *P* < 0.05.

### RNA-Seq.

Total RNA from WT and *Adap*^–/–^ septic mouse cells or LPS-stimulated WT and *Adap*^–/–^ PMs was extracted using TRIzol reagent (MilliporeSigma, T9424). RNA integrity and quantity were examined using an Agilent Bioanalyzer 2100 (Agilent Technologies). A sequencing library was prepared using the Hieff NGS Ultima Dual-mode mRNA Library Prep Kit (Yeasen, 12309ES) according to the manufacturer’s instructions. RNA-Seq was performed using the Gene Denovo Biotechnology Co. The DEGs between groups were quantified using DESeq2 (1.20.0). A 2-fold change and adjusted *P* < 0.05 were used for the identification of DEGs. Heatmaps were used to evaluate DEGs, and KEGG pathway analysis was performed to determine the significant functions and pathways of the DEGs. Normalized read counts of all expressed genes were subjected to GSEA. Bioinformatic analysis was performed using Omicsmart, a dynamic real-time interactive online platform for data analysis.

### Statistics.

All data were analyzed using GraphPad Prism 8 and are presented as mean ± SEM. For the comparison between 2 group means, a 2-tailed unpaired Student’s *t* test was used. For the comparison of more than 2 groups, a 1-way or 2-way ANOVA followed by Tukey’s or Šidák’s multiple-comparison test was used, as indicated specifically.

### Study approval.

All animal studies were approved by the Animal Ethics Committee of Soochow University (no. 202407A0072). This study involving the patient was approved by the IRB of the Second Affiliated Hospital of Soochow University and conducted in compliance with the Declaration of Helsinki (no. JD-LK2024075-I01).

### Data availability.

Values for all data points in graphs are reported in the Supporting Data Values file. RNA-Seq data have been deposited in the GEO database under accession nos. GSE281047, GSE281049, and GSE281052. All related data, code, and materials used in the analyses are available from the corresponding author upon reasonable request.

## Author contributions

HL conceived the study; HL designed experiments; PZ and XW performed experiments; PZ and HL analyzed and interpreted data; XY performed pathological examination and provided patient tissue samples; HL wrote and edited the manuscript with intellectual input from the other authors; and HL supervised and acquired funding for the study.

## Supplementary Material

Supplemental data

Unedited blot and gel images

Supporting data values

## Version 1 02/04/2025

In-Press Preview

## Version 2 03/24/2025

Electronic publication
