# Supplementary material for: Molecular control of PDPNhi macrophage subset induction by ADAP as a host defense in sepsis
Source: JCI Insight. 2025 Feb 4;10(6):e186456. doi: 10.1172/jci.insight.186456 (PMC11949065; doi:10.1172/jci.insight.186456)
Supplement: Supplemental data [file jciinsight-10-186456-s009.pdf]

Supplemental Figure 1

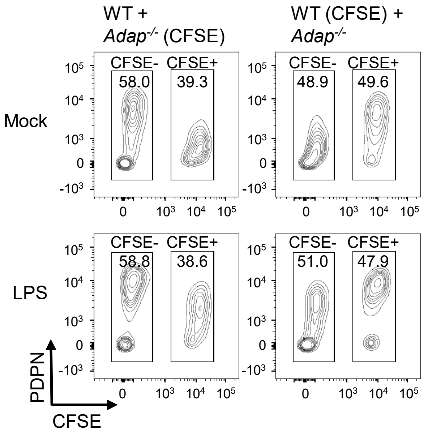

Supplemental Figure 1. The impairment of LPS-induced PDPN expression persisted in *Adap*$^{-/-}$ macrophages even when co-cultured with WT macrophages. WT or *Adap*$^{-/-}$ PMs were labeled with CFSE, and flow cytometry was applied to assess the expression of PDPN in the co-culture of WT and *Adap*$^{-/-}$ macrophages stimulated with or without LPS (100 ng/ml) for 24 h.

Supplemental Figure 2

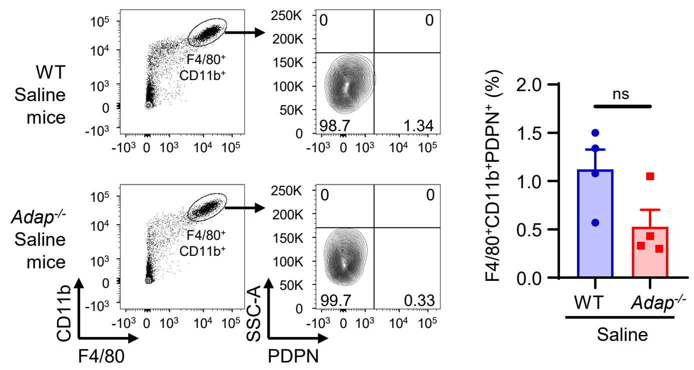

Supplemental Figure 2. Levels of PDPN^{hi} PMs are low in saline-treated WT and *Adap$^{-/-}$* mice. Peritoneal exudate cells were isolated from WT and *Adap$^{-/-}$* mice 18 h after saline injection, and CD11b^{+}F4/80^{+}PDPN^{hi} macrophages were analyzed by flow cytometry. Left panel: Representative contour plots showing the frequency of PDPN^{hi} macrophages in the peritoneal cavity of WT and *Adap$^{-/-}$* mice 18 h after saline injection. Right panel: Bar graph showing the percentage of PDPN^{hi} macrophages (n = 4 each, unpaired *t*-test).

Supplemental Figure 3

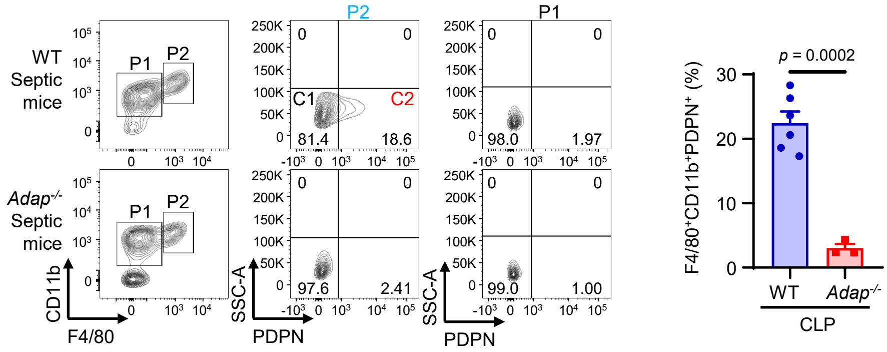

Supplemental Figure 3. The generation of PDPN^{hi} PMs is ADAP-dependent in the CLP-induced septic mouse model. WT and *Adap*$^{-/-}$ mice were subjected to CLP for 18 h, and peritoneal exudate cells were isolated to analyze CD11b^{+}F4/80^{+}PDPN^{hi} macrophages by flow cytometry. Left panel: Representative contour plots showing the frequency of PDPN^{hi} macrophages in the peritoneal cavity of WT and *Adap*$^{-/-}$ mice 18 h post-CLP. Right panel: Bar graph showing the percentage of PDPN^{hi} macrophages (WT, n = 6; *Adap*$^{-/-}$, n = 3; unpaired *t*-test).

Supplemental Figure 4

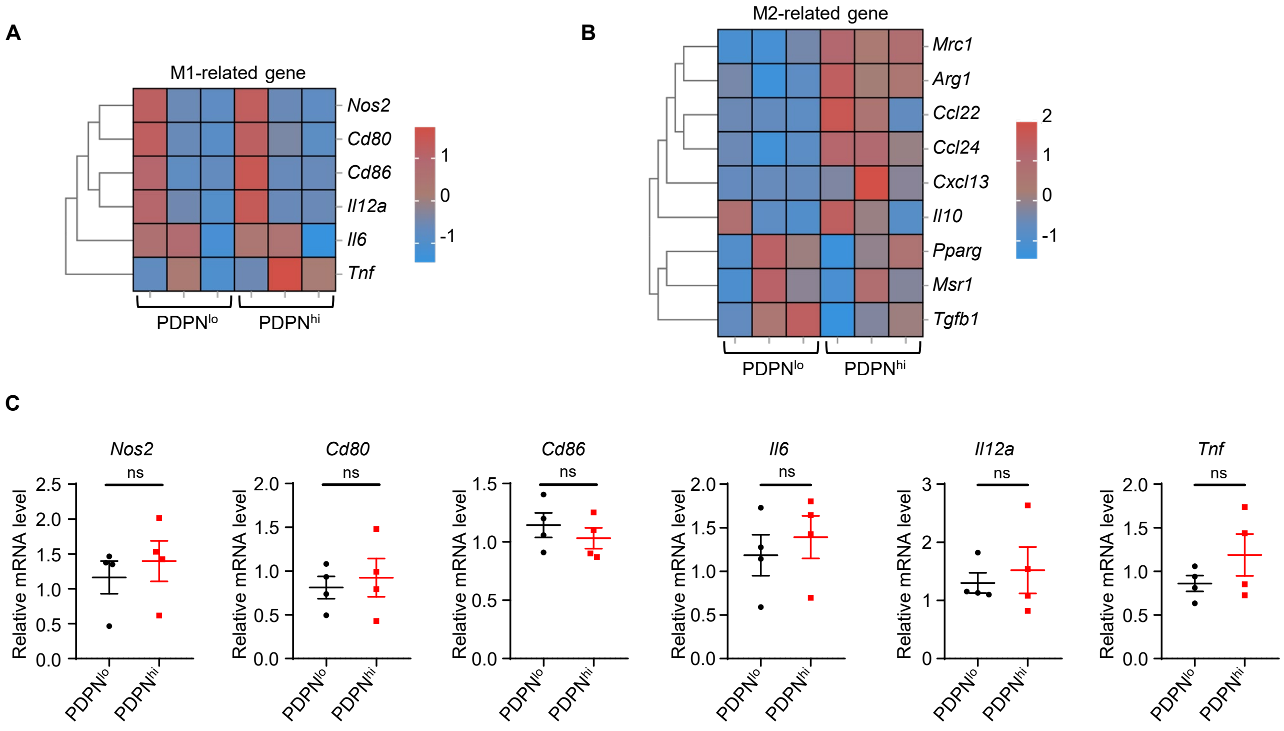

Supplemental Figure 4. The transcriptome of PDPN^{hi} PMs from WT septic mice exhibits an M2 phenotype. (**A** and **B**) Hierarchical clustering of macrophage polarization-related genes in RNA-seq analysis of PDPN^{hi} and PDPN^{lo} PMs sorted from the peritoneal cavity of WT mice 18 h post-injection of *E. coli* (2×10^7 CFU, i.p.). (**C**) The mRNA levels of M1 macrophage polarization markers in PDPN^{hi} and PDPN^{lo} PMs from WT septic mice were determined using RT-qPCR (n = 4 each, unpaired *t*-test). Relative mRNA levels were normalized to *Hprt*. ns, nonsignificant.

Supplemental Figure 5

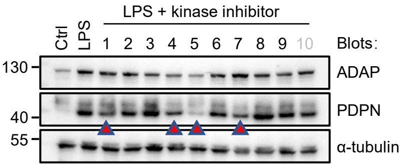

1: Linifanib (ABT-869)
2: Crizotinib (PF-02341066)
3: Saracatinib (AZD0530)
4: Dovitinib (TKI-258, CHIR-258)
5: Nilotinib (AMN-107)
6: Tozasertib (VX-680, MK-0457)
7: Axitinib
8: Y-27632 2HCl
9: Linsitinib (OSI-906)
10: Selumetinib (AZD6244)

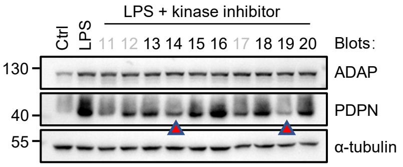

11: Enzastaurin (LY317615)
12: PD0325901
13: KU-55933 (ATM Kinase Inhibitor)
14: LY294002
15: Barasertib (AZD1152-HQPA)
16: Nintedanib (BIBF 1120)
17: AC480 (BMS-599626)
18: OSU-03012 (AR-12)
19: Everolimus (RAD001)
20: Afatinib (BIBW2992)

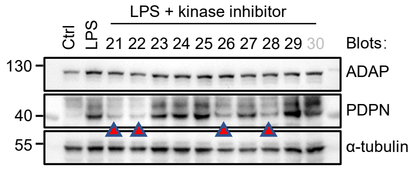

21: Gefitinib (ZD1839)
22: Rapamycin (Sirolimus)
23: SB202190 (FHPI)
24: BMS-754807
25: Roscovitine (Seliciclib,CYC202)
26: Regorafenib (BAY 73-4506)
27: Imatinib Mesylate (STI571)
28: Pictilisib (GDC-0941)
29: MK-2206 2HCl
30: BI 2536

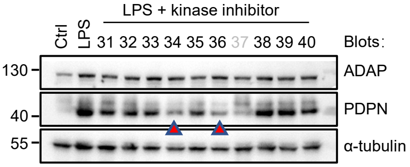

31: Alisertib (MLN8237)
32: Lenvatinib (E7080)
33: Bosutinib (SKI-606)
34: SU11274
35: Foretinib (GSK1363089)
36: ENMD-2076
37: CUDC-101
38: H 89 2HCl
39: CHIR-99021 (CT99021)
40: Thiazovivin

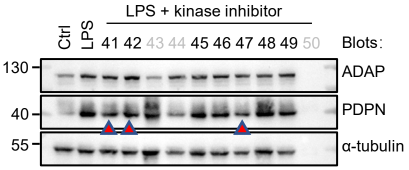

41: BIX 02189
42: AZD1480
43: NVP-BHG712
44: PD318088
45: AICAR (Acadesine)
46: PF-4708671
47: R406 (free base)
48: Vemurafenib (PLX4032, RG7204)
49: BS-181 HCl
50: Flavopiridol (Alvocidib)

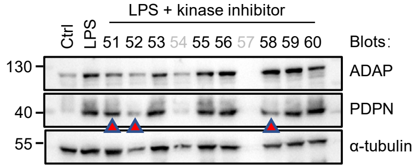

51: BX-795
52: AZD8055
53: Mubritinib (TAK 165)
54: Quizartinib (AC220)
55: Tie2 kinase inhibitor
56: 3-Methyladenine (3-MA)
57: Dinaciclib (SCH727965)
58: AZD4547
59: Quercetin
60: Crenolanib (CP-868596)

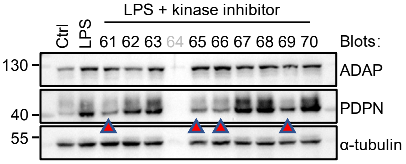

61: Trametinib (GSK1120212)
62: Degrasyn (WP1130)
63: Daphnetin
64: CUDC-907
65: Buparlisib (BKM120, NVP-BKM120)
66: Ibrutinib (PCI-32765)
67: Sotrastaurin
68: Tofacitinib (CP-690550) Citrate
69: PF-3758309
70: RKI-1447

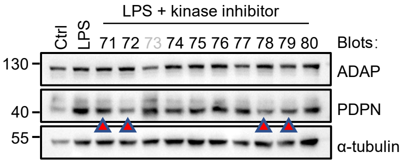

71: Fingolimod (FTY720) HCl
72: Duvelisib (IPI-145, INK1197)
73: VE-822
74: 10058-F4
75: PF-562271
76: Go 6983
77: Ceritinib (LDK378)
78: Spebrutinib (CC-292, AVL-292)
79: BI-D1870
80: GSK2334470

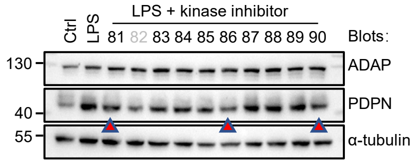

81: SKI II
82: JNK-IN-8
83: PP2
84: GNE-0877
85: Ulixertinib (BVD-523, VRT752271)
86: Sorafenib
87: XMD8-92
88: Anacardic Acid
89: KN-93 Phosphate
90: LDN-214117

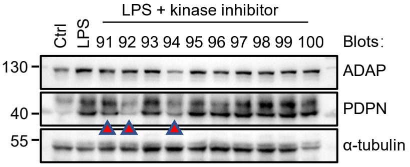

91: SU6656
92: CEP-32496
93: PFK15
94: URMC-099
95: Entrectinib (RXDX-101)
96: Dasatinib
97: D 4476
98: DASA-58
99: NSC 23766
100: SNS-314 Mesylate

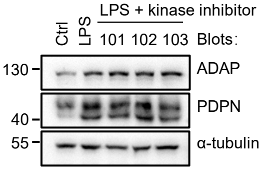

101: Perifosine (KRX-0401)
102: Palbociclib (PD-0332991) HCl
103: Dorsomorphin (Compound C) 2HCl

▲ : kinase inhibitors that block LPS-induced PDPN upregulation

Light-font color : kinase inhibitors that were toxic to cells

Supplemental Figure 5. Effects of kinase inhibitors on LPS-induced PDPN expression in macrophages. Western blot analysis of protein expression in PMs after LPS stimulation (100 ng/ml) in the absence or presence of 103 kinase inhibitors (1 μM) for 24 h. Triangle: kinase inhibitors that block LPS-induced PDPN upregulation. Light-font color: kinase inhibitors that were toxic to cells.

Supplemental Figure 6

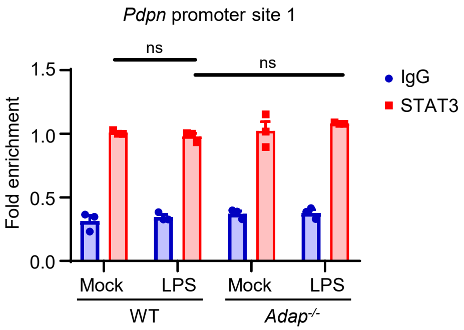

Supplemental Figure 6. LPS fails to stimulate the binding of STAT3 to site 1 of the *Pdpn* promoter in macrophages. CUT & RUN-qPCR analyses were performed to examine the enrichment of the *Pdpn* promoter (site 1) by the anti-STAT3 antibody in PMs that were either untreated or treated with LPS (100 ng/ml, 1 h) (n = 3 each, two-way ANOVA, Tukey's multiple comparison).

Supplemental Figure 7

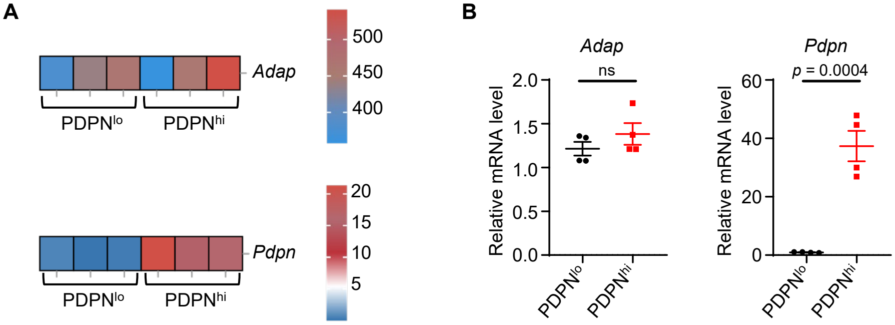

Supplemental Figure 7. ADAP expression remains unchanged between PDPN^{hi} and PDPN^{lo} PMs in WT septic mice. (**A**) Hierarchical clustering of *Adap* and *Pdpn* in RNA-seq analysis of PDPN^{hi} and PDPN^{lo} PMs sorted from the peritoneal cavity of WT mice 18 h post-injection of *E. coli* (2×10^7 CFU, i.p.). (**B**) The mRNA levels of *Adap* and *Pdpn* in PDPN^{hi} and PDPN^{lo} PMs from WT septic mice were determined using RT-qPCR (n = 4 each, unpaired t-test). Relative mRNA levels were normalized to *Hprt*. ns, nonsignificant.

Supplemental Table 1: Kinase Inhibitors that block LPS-induced PDPN upregulation

Number	Name	Target	Pathway
1	Linifanib (ABT-869)	CSF-1R,PDGFR,VEGFR	Protein Tyrosine Kinase
4	Dovitinib (TKI-258, CHIR-258)	C-Kit,FGFR, FLT3,PDGFR,VEGFR	Angiogenesis
5	Nilotinib (AMN-107)	Bcr-Abl	Angiogenesis
7	Axitinib	c-Kit,PDGFR,VEGFR	Protein Tyrosine Kinase
14	LY294002	Autophagy,PI3K	PI3K/Akt/mTOR
19	Everolimus (RAD001)	mTOR	PI3K/Akt/mTOR
21	Gefitinib (ZD1839)	EGFR	Protein Tyrosine Kinase
22	Rapamycin (Sirolimus)	mTOR	PI3K/Akt/mTOR
26	Regorafenib (BAY 73-4506)	c-RET,VEGFR	Protein Tyrosine Kinase
28	Pictilisib (GDC-0941)	PI3K	PI3K/Akt/mTOR
34	SU11274	c-Met	Protein Tyrosine Kinase
36	ENMD-2076	Aurora Kinase,FLT3, VEGFR	Angiogenesis
41	BIX 02189	MEK	MAPK
42	AZD1480	JAK	JAK/STAT
47	R406 (free base)	Syk	Angiogenesis
51	BX-795	IκB/IKK,PDK	PI3K/Akt/mTOR
52	AZD8055	mTOR	PI3K/Akt/mTOR
58	AZD4547	FGFR	Angiogenesis
61	Trametinib (GSK1120212)	MEK	MAPK
65	Buparlisib (BKM120, NVP-BKM120)	PI3K	PI3K/Akt/mTOR
66	Ibrutinib (PCI-32765)	BTK	Angiogenesis
69	PF-3758309	PAK	Cytoskeletal Signaling
71	Fingolimod (FTY720) HCl	S1P Receptor	GPCR & G Protein
72	Duvelisib (IPI-145, INK1197)	PI3K	Angiogenesis
78	Spebrutinib (CC-292, AVL-292)	BTK	Angiogenesis
79	BI-D1870	S6 Kinase	PI3K/Akt/mTOR
81	SKI II	S1P Receptor	GPCR & G Protein
86	Sorafenib	Raf	MAPK
90	LDN-214117	TGF-beta/Smad	TGF-beta/Smad
91	SU6656	Src	Angiogenesis
92	CEP-32496	CSF-1R,Raf	MAPK
94	URMC-099	LRRK2, mixed lineage kinase (MLK) inhibitor	Autophagy

Supplemental Table 2: Sequences of RT-qPCR primers involved in the study

Name	Forward 5'-3'	Reverse 5'-3'
hADAP	CCAACCACCATTGCCAGCATCT	CATCAGAGTGCGTGACACCATC
hYWHAZ	ACCGTTACTTGGCTGAGGTTGC	CCCAGTCTGATAGGATGTGTTGG
mIL-1β	TCCAGGATGAGGACATGAGCAC	GAACGTCACACACCAGCAGGTTA
mIL-6	AGTTGCCTTCTTGGGACTGA	TCCACGATTTCCCAGAGAAC
mTNF-α	CGGTGCCTATGTCTCAGCCT	GAGGGTCTGGGCCATAGAAC
mGAPDH	GGGTCCCAGCTTAGGTTCATC	ACTGTGCCGTTGAATTTGCC
mADAP	AAAAGTGGGGAGCGAGAGAT	CCAGGTAACTCCGAAGGACA
mPDPN	ACAACCACAGGTGCTACTGGAG	GTTGCTGAGGTGGACAGTTCCT
mHPRT	GGTGAAAAGGACCTCTCGAA	AGTCAAGGGCATATCCAACA
mNOS2	GAGACAGGGAAGTCTGAAGCAC	CCAGCAGTAGTTGCTCCTCTTC
mCD80	CCTCAAGTTTCCATGTCCAAGGC	GAGGAGAGTTGTAACGGCAAGG
mCD86	ACGTATTGGAAGGAGATTACAGCT	TCTGTCAGCGTTACTATCCCGC
mIL12a	ACGAGAGTTGCCTGGCTACTAG	CCTCATAGATGCTACCAAGGCAC
mMRC1	GTTCACCTGGAGTGATGGTTCTC	AGGACATGCCAGGGTCACCTTT
mARG1	CATTGGCTTGCGAGACGTAGAC	GCTGAAGGTCTCTTCCATCACC
mMSR1	CGCACGTTCAATGACAGCATCC	GCAAACACAAGGAGGTAGAGAGC
mPPAR-γ	GTACTGTCGGTTTCAGAAGTGCC	ATCTCCGCCAACAGCTTCTCCT
mTGF-β	TGATACGCCTGAGTGGCTGTCT	CACAAGAGCAGTGAGCGCTGAA
mIL10	CGGGAAGACAATAACTGCACCC	CGGTTAGCAGTATGTTGTCCAGC
mCCL24	ATTCCAGAAAACCGAGTGGTTAGC	GCATCCAGTTTTTGTATGTGCCTC
mCCL22	GTGGAAGACAGTATCTGCTGCC	AGGCTTGCGGCAGGATTTTGAG
mCXCL13	CATAGATCGGATTCAAGTTACGCC	GTAACCATTTGGCACGAGGATTC
mCD36	GGACATTGAGATTCTTTTCCTCTG	GCAAAGGCATTGGCTGGAAGAAC
mMARCO	ATGGCACCAAGGGAGACAAAGG	GCCTGGTTTTCCAGCATCACCT
