# Supplementary material for: Molecular control of PDPNhi macrophage subset induction by ADAP as a host defense in sepsis
Source: JCI Insight. 2025 Feb 4;10(6):e186456. doi: 10.1172/jci.insight.186456 (PMC11949065; doi:10.1172/jci.insight.186456)

Full unedited gel for Figure 1

Figure 1D

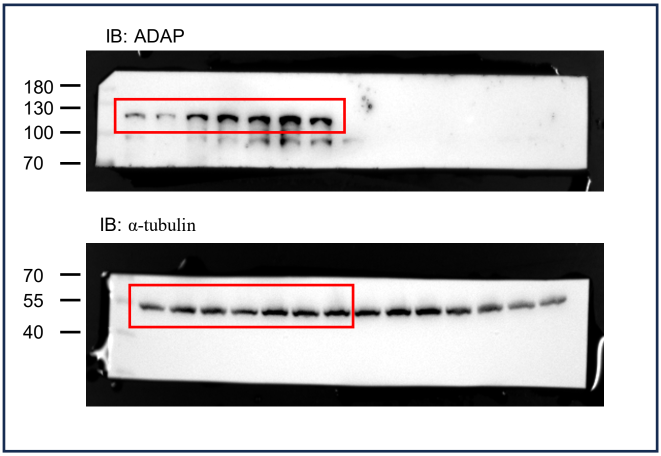

Figure 1E left panel

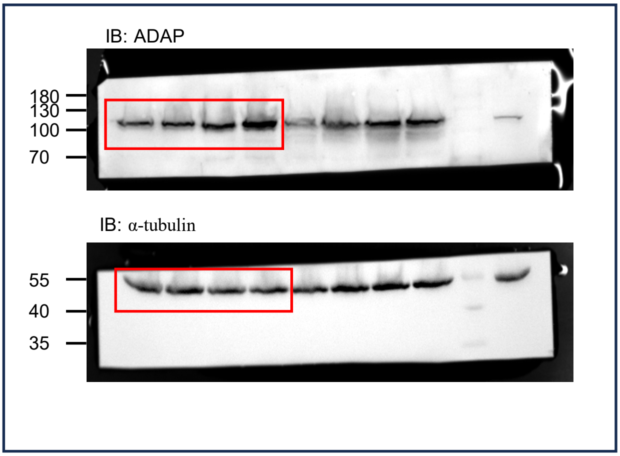

Figure 1E middle panel

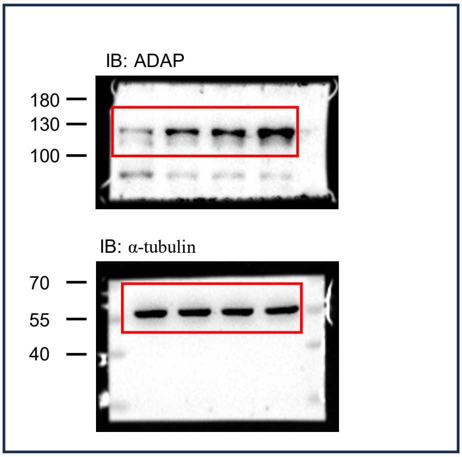

Figure 1E right panel

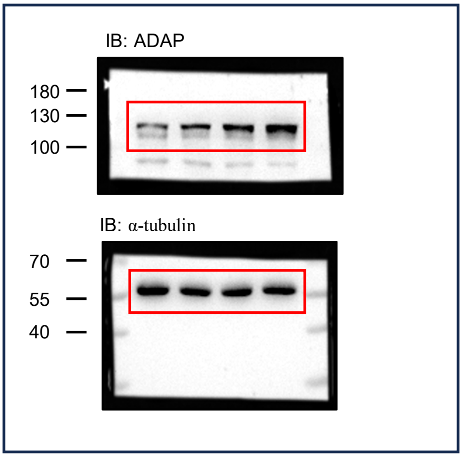

Figure 1F

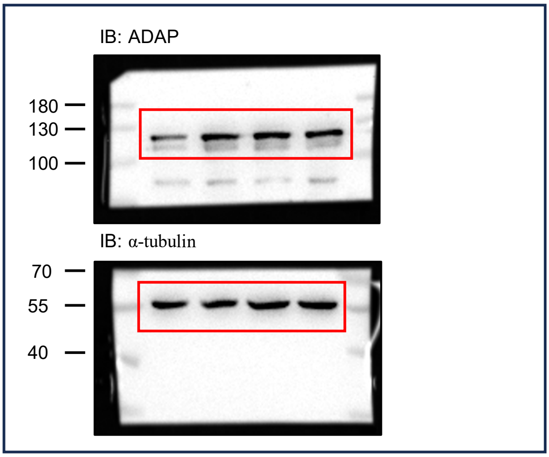

Full unedited gel for Figure 3

Figure 3D upper panel

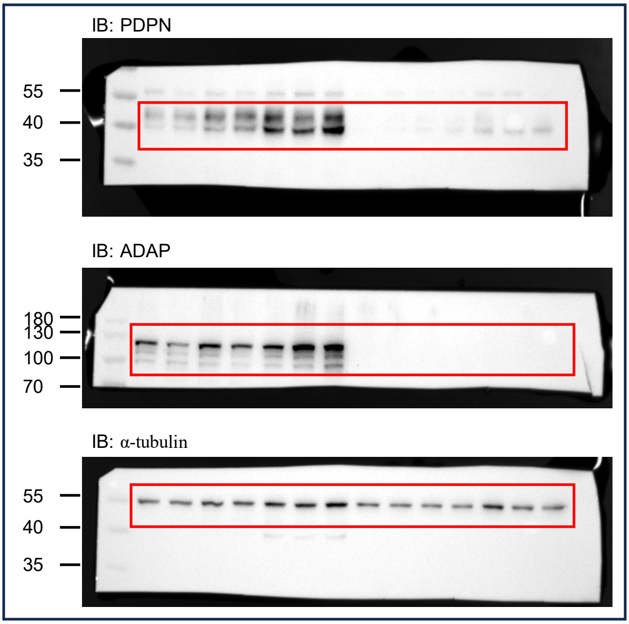

Figure 3D lower panel

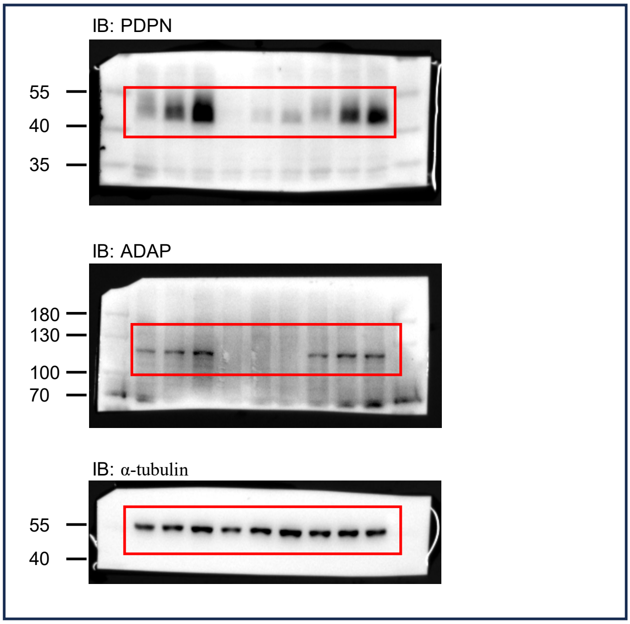

Figure 3G

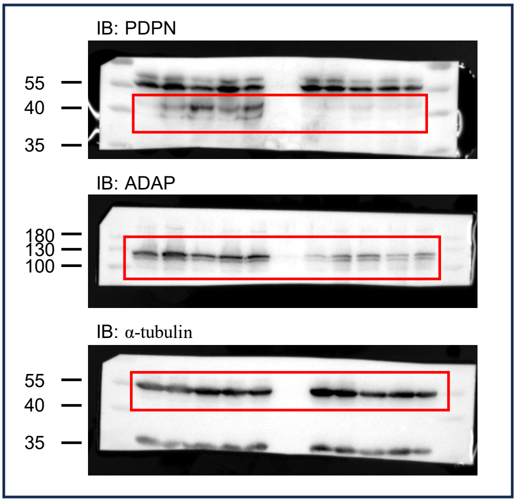

Figure 3I

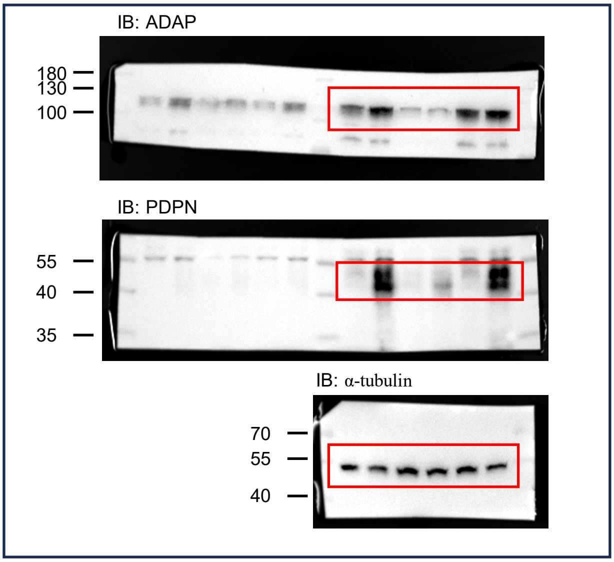

Full unedited gel for Figure 6

Figure 6A

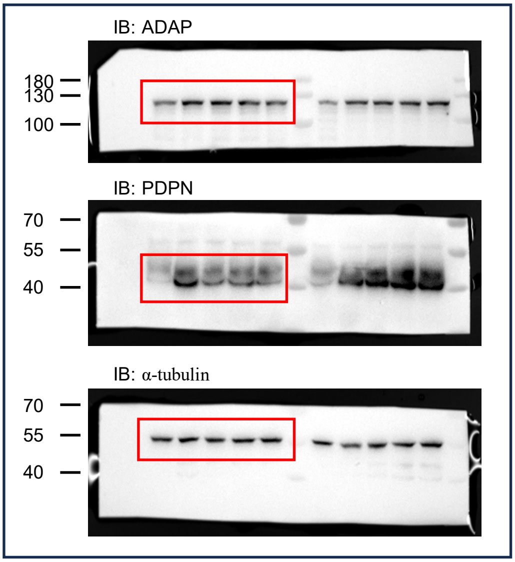

Figure 6C

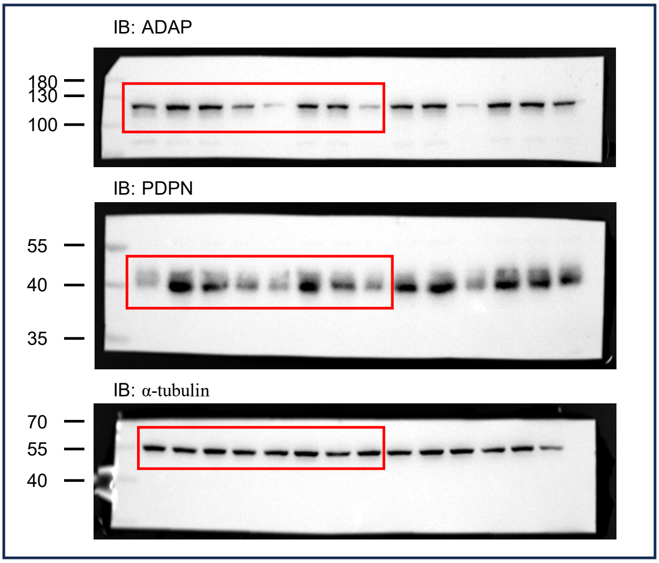

Figure 6D

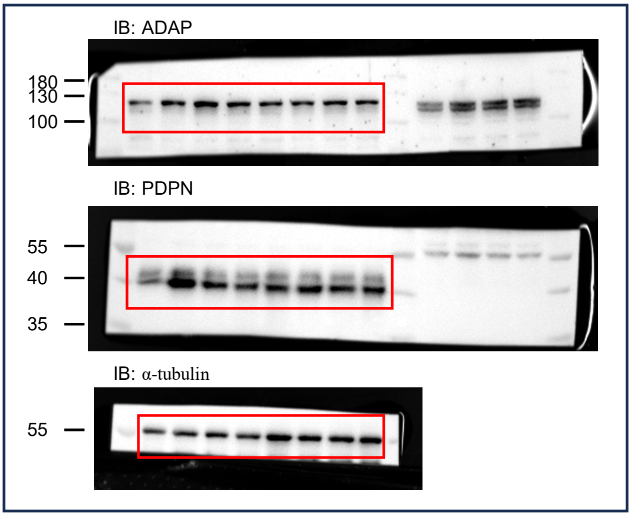

Figure 6E

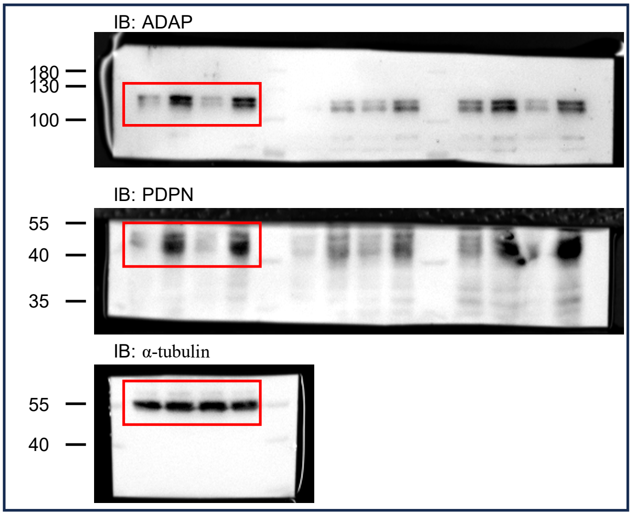

Figure 6G

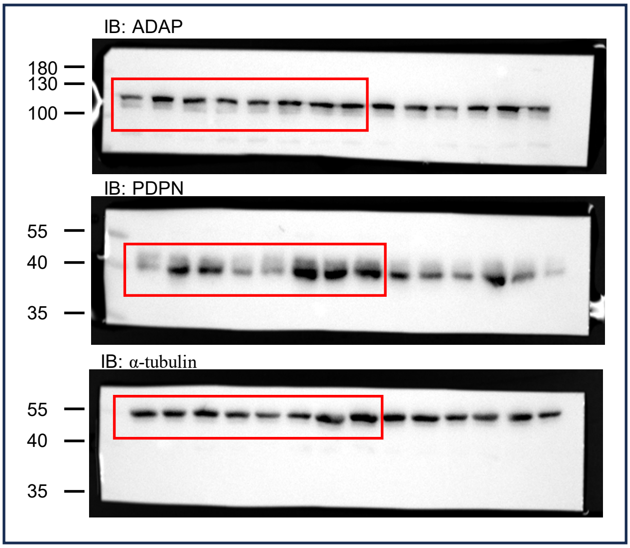

Figure 6I

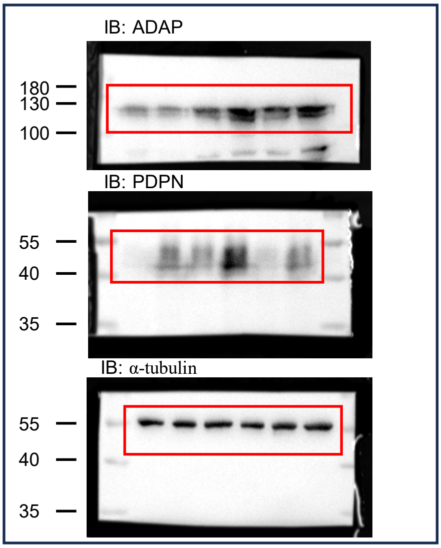

Full unedited gel for Figure 6

Figure 6J

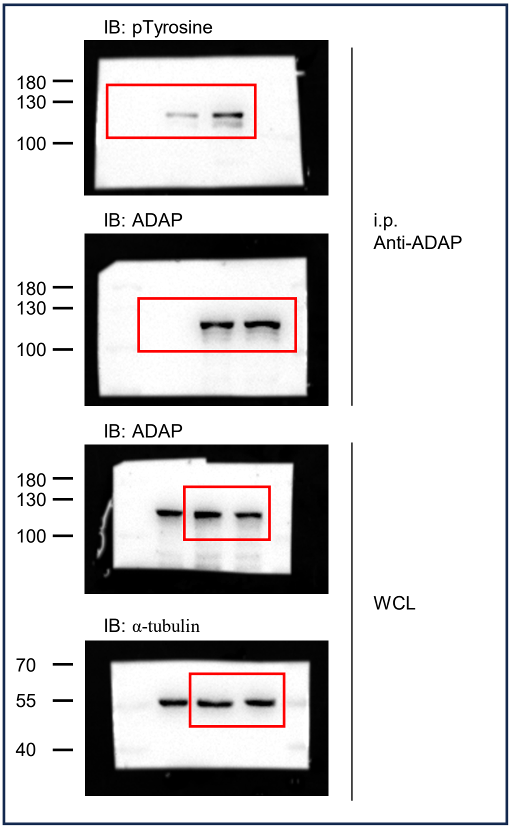

Full unedited gel for Figure 7

Figure 7A

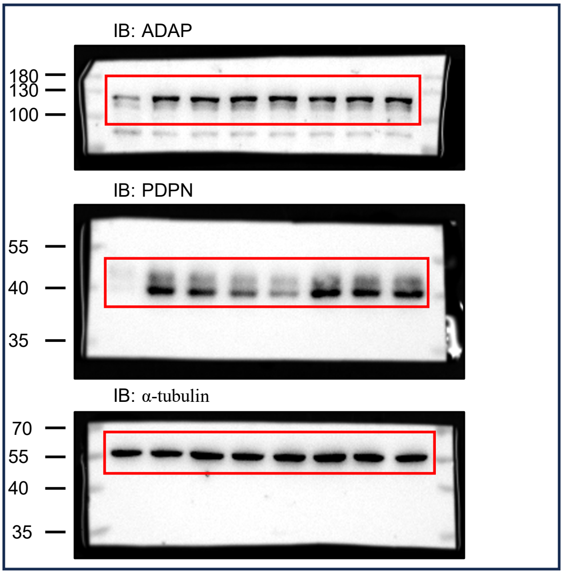

Figure 7D

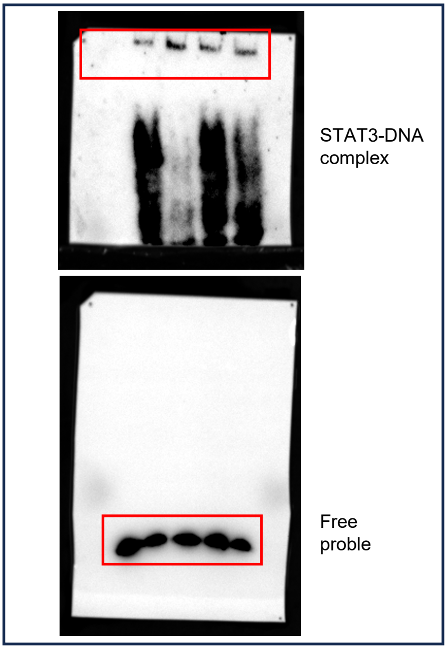

Figure 7F

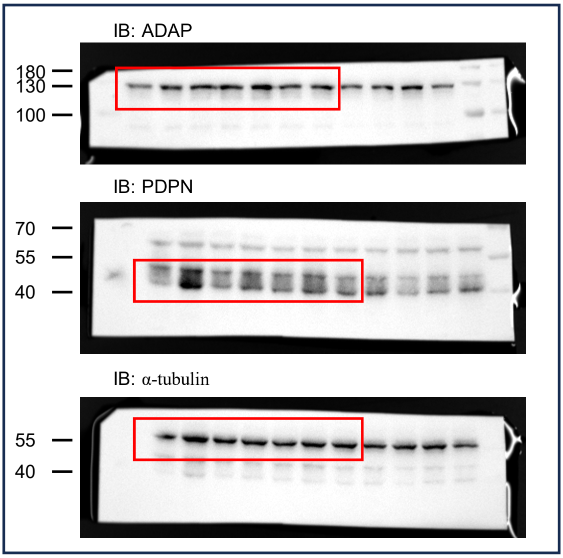

Figure 7G

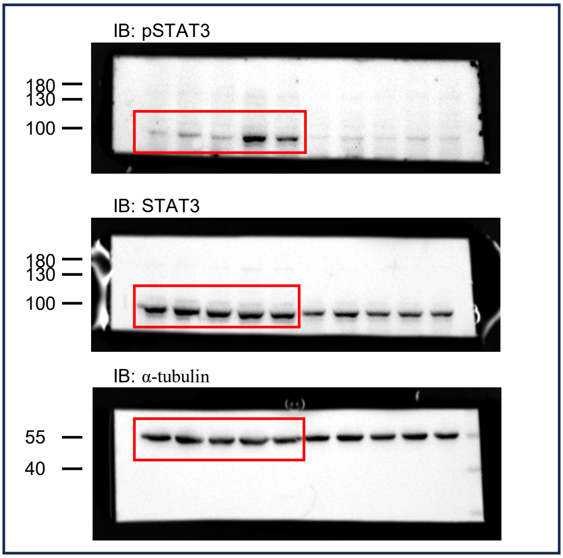

Figure 7H

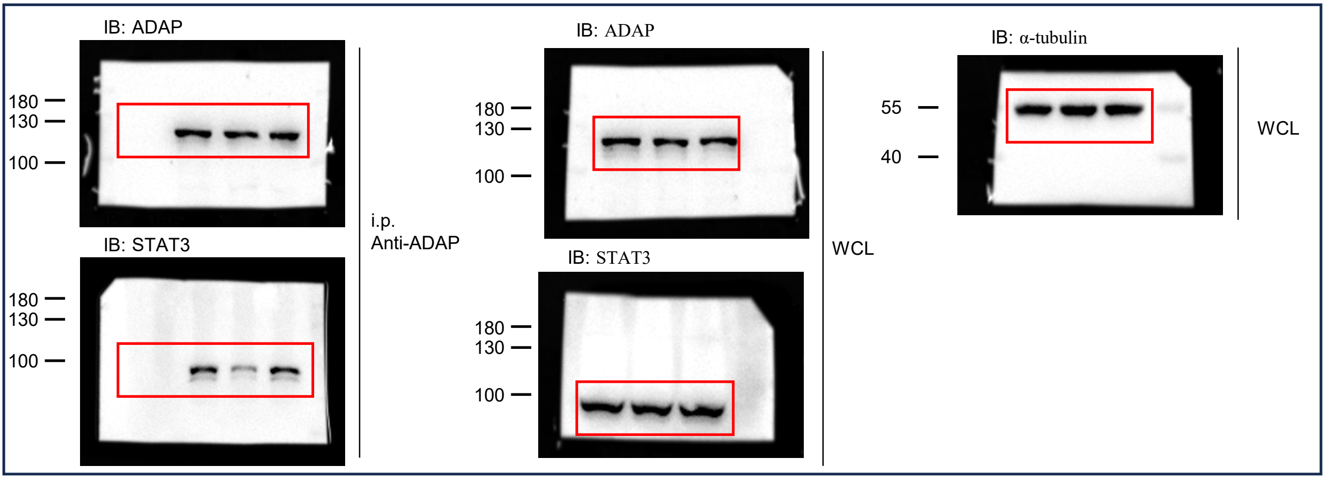

Full unedited gel for Figure 7

Figure 7I

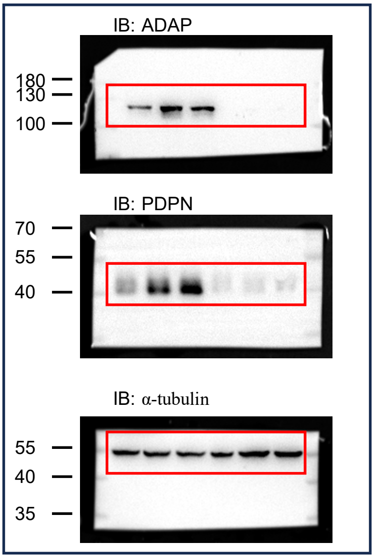

Full unedited gel for Supplement Figure 4

Figure S4 (inhibitor: 1-10)

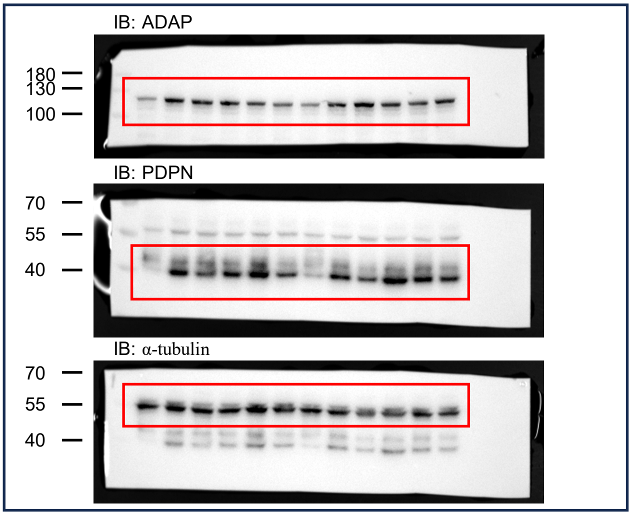

Figure S4 (inhibitor: 11-20)

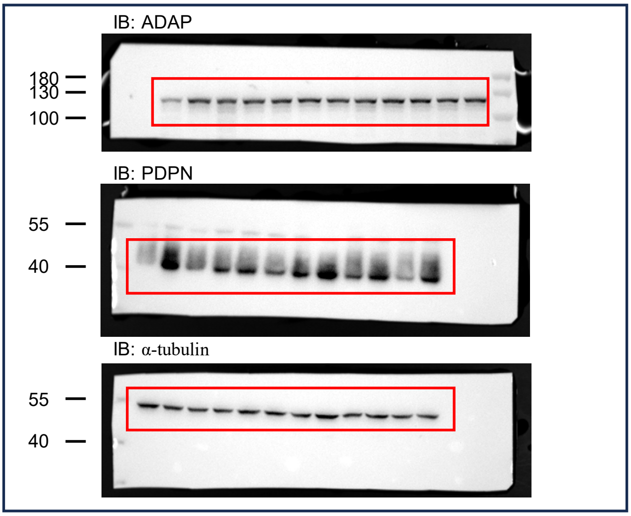

Figure S4 (inhibitor: 21-30)

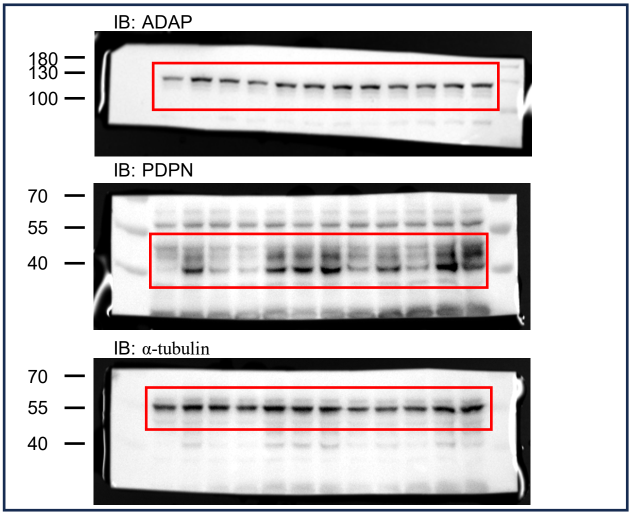

Figure S4 (inhibitor: 31-40)

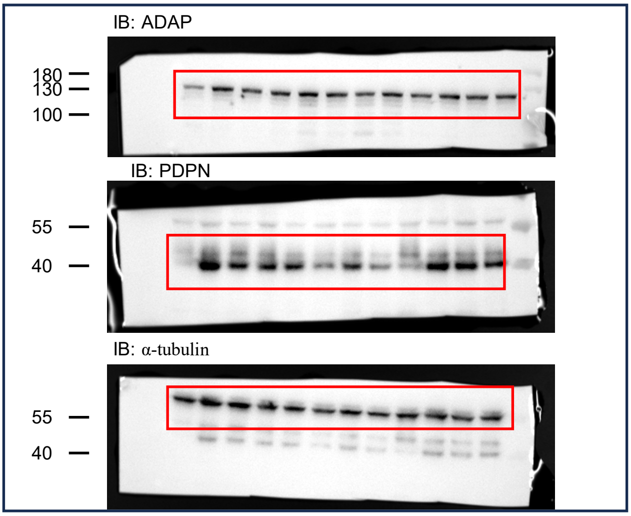

Figure S4 (inhibitor: 41-50)

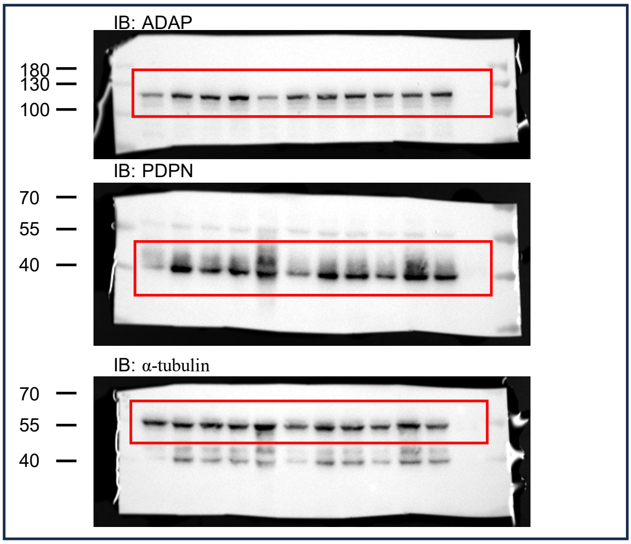

Figure S4 (inhibitor: 51-60)

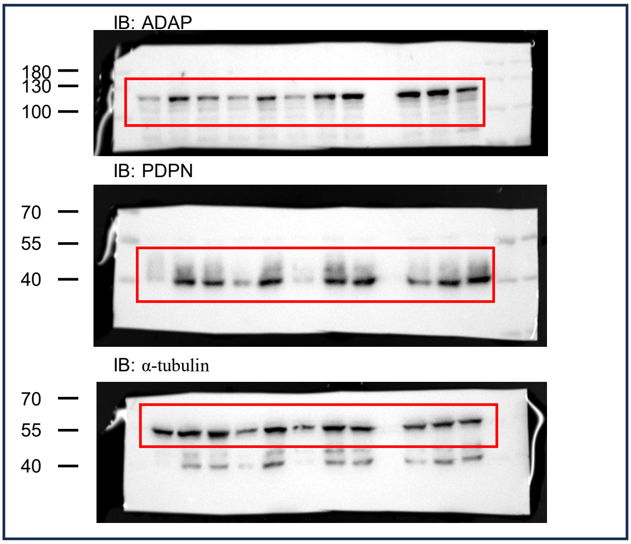

Full unedited gel for Supplement Figure 4

Figure S4 (inhibitor: 61-70)

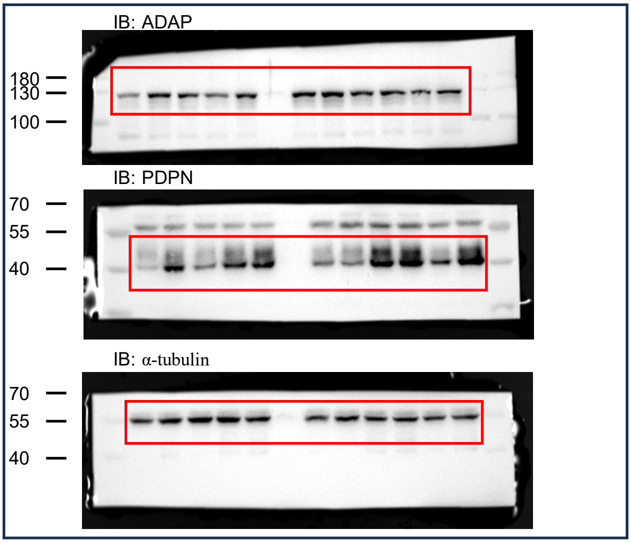

Figure S4 (inhibitor: 71-80)

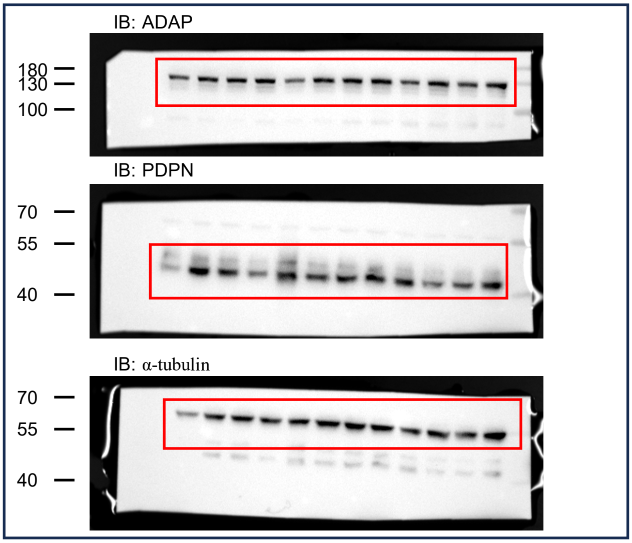

Figure S4 (inhibitor: 81-90)

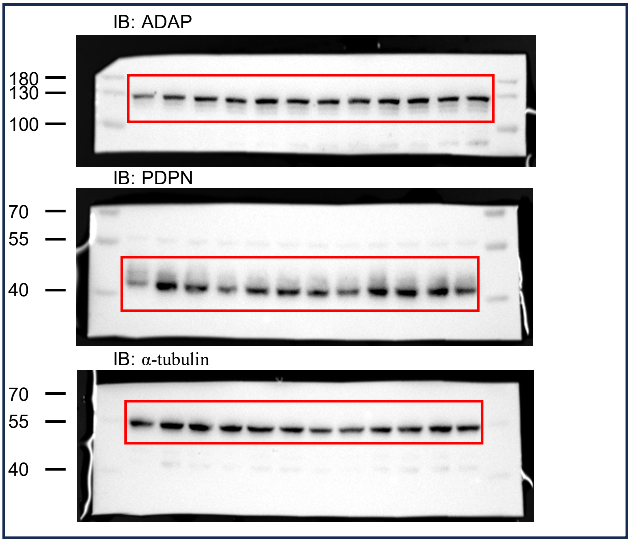

Figure S4 (inhibitor: 91-100)

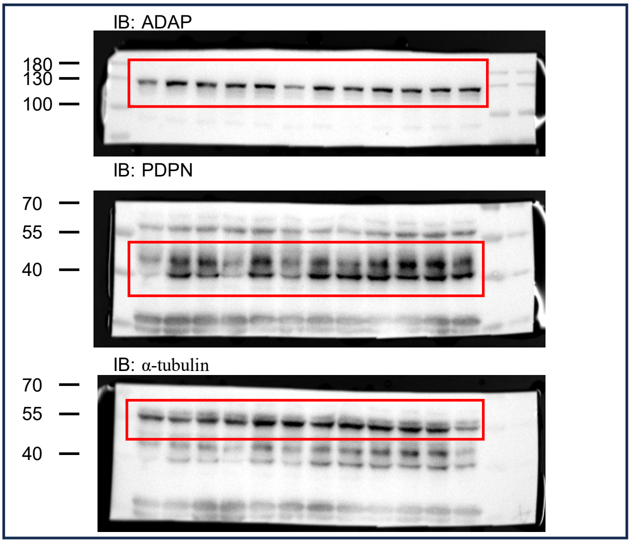

Figure S4 (inhibitor: 101-103)

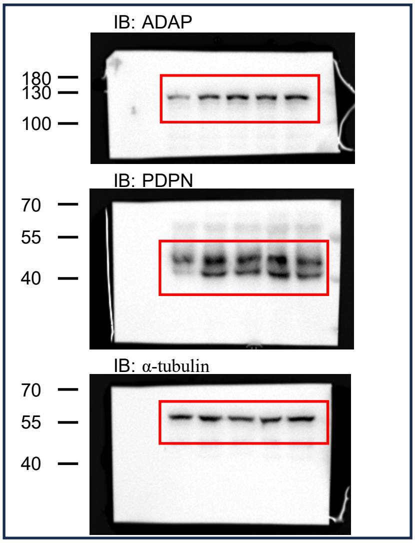

Supplement: Unedited blot and gel images [file jciinsight-10-186456-s010.pdf]
